# A Genomic Approach to Unravel Host-Pathogen Interaction in Chelonians: The Example of *Testudinid Herpesvirus 3*

**DOI:** 10.1371/journal.pone.0134897

**Published:** 2015-08-05

**Authors:** Francesco C. Origgi, Marco Tecilla, Paola Pilo, Fabio Aloisio, Patricia Otten, Lisandra Aguilar-Bultet, Ursula Sattler, Paola Roccabianca, Carlos H. Romero, David C. Bloom, Elliott R. Jacobson

**Affiliations:** 1 Centre for Fish and Wildlife Health (FIWI), Vetsuisse Faculty, University of Bern, Bern, Switzerland; 2 Department of veterinary sciences and public health (DIVET) Universita’ degli studi di Milano, Milano, Italy; 3 Institute of Veterinary Bacteriology, Vetsuisse Faculty, University of Bern, Bern, Switzerland; 4 IDEXX Laboratories Italia S.r.l., Milano, Italy; 5 Fasteris SA, Geneva, Switzerland; 6 Graduate School for Cellular and Biomedical Sciences, University of Bern, Bern Switzerland; 7 Department of Infectious Diseases and Pathology, College of Veterinary Medicine, University of Florida, Gainesville, Florida, United States of America; 8 Department of Molecular Genetics & Microbiology, College of Medicine, University of Florida, Gainesville, Florida, United States of America; 9 Department of Small Animal Clinical Sciences, College of Veterinary Medicine, University of Florida, Gainesville, Florida, United States of America; University of Minnesota, UNITED STATES

## Abstract

We report the first *de novo* sequence assembly and analysis of the genome of *Testudinid herpesvirus 3* (TeHV3), one of the most pathogenic chelonian herpesviruses. The genome of TeHV3 is at least 150,080 nucleotides long, is arranged in a type D configuration and comprises at least 102 open reading frames extensively co-linear with those of *Human herpesvirus 1*. Consistently, the phylogenetic analysis positions TeHV3 among the *Alphaherpesvirinae*, closely associated with *Chelonid herpesvirus 5*, a *Scutavirus*. To date, there has been limited genetic characterization of TeHVs and a resolution beyond the genotype was not feasible because of the lack of informative DNA sequences. To exemplify the potential benefits of the novel genomic information provided by this first whole genome analysis, we selected the glycoprotein B (gB) gene, for detailed comparison among different TeHV3 isolates. The rationale for selecting gB is that it encodes for a well-conserved protein among herpesviruses but is coupled with a relevant antigenicity and is consequently prone to accumulate single nucleotide polymorphisms. These features were considered critical for an ideal phylogenetic marker to investigate the potential existence of distinct TeHV3 genogroups and their associated pathology. Fifteen captive tortoises presumptively diagnosed to be infected with TeHVs or carrying compatible lesions on the basis of either the presence of intranuclear inclusions (presumptively infected) and/or diphtheronecrotic stomatitis-glossitis or pneumonia (compatible lesions) were selected for the study. Viral isolation, TeHV identification, phylogenetic analysis and pathological characterization of the associated lesions, were performed. Our results revealed 1) the existence of at least two distinct TeHV3 genogroups apparently associated with different pathologies in tortoises and 2) the first evidence for a putative homologous recombination event having occurred in a chelonian herpesvirus. This novel information is not only fundamental for the genetic characterization of this virus but is also critical to lay the groundwork for an improved understanding of host-pathogen interactions in chelonians and contribute to tortoise conservation.

## Introduction

Herpesviruses have been reported as significant pathogens of snakes [1, 2], lizards [3–10] and alligators [11, 12], however, more herpesviruses have been detected in chelonians than in any other reptilian taxa [13]. Presently, chelonian herpesviruses have been detected and/or isolated from both turtles and tortoises [14]. The first detection of a chelonian herpesvirus dates back to the mid 70’s with the discovery of *Chelonid herpesvirus 1* (ChHV1), known as “Gray patch disease-associated herpesvirus”. The disease consisted in a multifocal to coalescent necrotizing and ulcerative dermatitis observed mainly in young green turtles (*Chelonia mydas*) [15]. *Chelonid herpesvirus 2* and *3* (ChHV2, 3) were detected in fresh water turtles and more specifically in Pacific pond turtles (*Clemmys marmorata*-ChHV2) [16] and in painted turtles (*Chrysemis picta*-ChHV3) [17] with necrotizing hepatitis. *Chelonid herpesvirus 4* (ChHV4) was detected in Argentine tortoises (*Geochelone chilensis*-ChHV4) with glossitis, rhinitis and pharyngitis [18]. Additional four herpesviruses were either detected or isolated from tortoises and were named *Testudinid herpesviruses* (TeHV1, 2, 3 and 4). TeHV1, 2 and 3 were associated with pathology mainly in tortoises of the genus *Testudo* (TeHV1 and 3) and in desert tortoises (*Gopherus agassizii*) (TeHV2) [14], whereas TeHV4 was detected in a clinically healthy bowsprit tortoise (*Chersina angulata*) [14, 19–21]. *Chelonid herpesvirus 5* and *6* (ChHV5, 6) are two other sea turtle herpesviruses associated with a debilitating neoplastic disease called fibropapillomatosis (mainly in green and loggerhead-*Caretta caretta*-sea turtles) and with a multisystemic disease (in green turtles), respectively [22–25]. Two additional herpesviruses have been discovered in sea turtles and have been named loggerhead genital herpesvirus and the loggerhead cutaneous herpesvirus and they are responsible for multisystemic lesions in loggerhead sea turtles [13]. In the last few years an increasing number of novel herpesviruses have been detected in fresh water turtles. These include a herpesvirus infecting the Australian Krefft’s river turtles (*Emydura macquarii kreftii*) associated with proliferative and ulcerative tegument lesions [26] and *Pelomedusid herpesvirus 1* in a clinically healthy West African mud turtle (*Pelusios castaneous*) [27]. *Emydid herpesvirus 1* is a novel chelonian herpesvirus detected in an eastern river cooter (*Pseudemys concinna concinna*) with viral inclusions in the hepatocytes [28] and in both diseased (with pneumonia, hepatitis and splenitis) and clinically healthy northern map turtles (*Graptmeys geographica*) along with asymptomatic painted turtles [29]. *Glyptemis herpesvirus 1* and *Glyptemys herpesvirus 2* were recently detected in asymptomatic bog (*Glyptemis muhlenbergii*) and wood turtles (*Glyptemis insculpta*), respectively, whereas *Emydid herpesvirus 2* was found in bog turtles and in a spotted turtle [30]. At least two herpesviruses have been detected in box turtles. *Terrapene herpesvirus 1* was detected in captive eastern box turtles (*Terrapene carolina carolina*) with stomatitis and glossitis [31], whereas *Terrapene herpesvirus 2* was recently identified in an eastern box turtle coinfected with a spirorchid trematode and with papillomatous growths [32].

Despite the existence of all the chelonian herpesviruses described above, to date the only genome sequenced is that of ChHV5, a *Scutavirus* [22]. Only a few hundred nucleotides (nt) of DNA sequence belonging to the DNA polymerase (DNApol) gene have been determined for other herpesviruses infecting reptiles [33], accounting for less than 0.01% of their entire predicted genomes. These short sequences have been used to unravel the phylogenetic relatedness of reptilian herpesviruses. However, the relatively high conservation of the DNApol gene among herpesviruses, and more specifically of the selected PCR-amplified-region, has hampered any higher resolution regarding diversity of these viruses beyond the genotype.

*Testudinid herpesviruses* are important pathogens that have been detected in the chelonian family *Testudinidae* (tortoises) [14, 19–21]. Of the three genotypes, associated with obvious pathology, TeHV1 has been detected most frequently in Horsfield’s tortoises (*Testudo-Agryonemis horsfieldii*) having stomatitis and glossitis [14]. TeHV2 has been identified in North American desert tortoises [20, 21]. Severe stomatitis, glossitis and pneumonia have been observed in a TeHV2-infected captive desert tortoise along with pneumonia [21]. TeHV2 has been shown to serologically cross-react by ELISA with TeHV3 suggesting the existence of similar antigenic epitopes in the two distinct genotypes [20, 21]. Consistently, the partial sequence of the TeHV2 ribonucleotide reductase gene (large subunit), a conserved gene in herpesviruses, shared 79% identity with the homologous TeHV3 gene [21]. TeHV3 is considered the most pathogenic of the known TeHVs and it has been detected in several species of tortoises associated with stomatitis and glossitis although it appears to be overrepresented in Greek tortoises (*Testudo graeca*) [14]. The pathology of TeHV3 has been thoroughly characterized in a transmission study in Greek tortoises infected with the type-strain 1976/98 with the virus inducing a disease whose severity was viral-load-dependent [34]. The classic clinical signs associated with the disease including stomatitis, mono- and bilateral recurrent conjunctivitis and oral discharge were reproduced in the experimentally infected tortoises [34]. In this study, the virus showed prominent neurotropism and indirect evidence of latency. Furthermore the severity of the disease appears to vary with the species of tortoise infected. For instance, TeHV3 infection in the Greek tortoise is generally characterized by low mortality, whereas a severe, acute to sub-acute disease with high mortality has been observed in Hermann’s tortoises (*Testudo hermanni*) [14].

Although numerous serological and molecular tests have been developed to diagnose exposure and infection with TeHV3 [33, 34–38], none of these tests can predict the virulence of the strain or whether one or several strains are involved within a single outbreak. To better understand the biology of this virus, we decided to sequence the complete genome of the TeHV3 type-strain 1976/96 [34, 36], the best characterized TeHV3 strain to date, to provide a complete reference genome sequence for TeHV3. Knowledge of this complete sequence in synthesis with further phylogenetic and experimental data would facilitate a better understanding of the host-pathogen relationship in chelonians and the development of more refined diagnostic tests contributing to predict the outcome of an outbreak. In addition, in this study we selected the sequence of the glycoprotein B (gB), gene as a tool to perform a higher resolution phylogenetic analysis on TeHV3 strains. The choice of the gB gene over other TeHV3 genes was based on its key features as an ideal phylogenetic marker including conservation among herpesviruses and sequence variability secondary to its antigenicity [39, 40]. These characteristics are expected to refine the resolution power of the viral DNApol sequence since the protein encoded by this gene is expected to be under a negative evolutionary pressure because of strong functional constraint. As we will show, the gB sequence allowed us to separate TeHV3 strains into at least two distinct genogroups. Moreover, we present preliminary data consistent with distinct genogroup-associated pathology in naturally and experimentally infected tortoises. We provide the first evidence of homologous recombination in a reptilian herpesvirus with indirect proof of a single tortoise infected with multiple strains of TeHV3. These new data will be fundamental for further investigations on host-pathogen interactions in chelonians and for tortoise conservation around the world.

In this manuscript we also propose to uniform the nomenclature of the TeHVs strains by presenting in the following order: the abbreviation of the country where the strain originated, followed by the original number assigned to the strain and the last two digits corresponding to the year of isolation. According to this procedure, the type strain 1976/96 will be renamed as (TeHV3)-US1976/98. All the other TeHVs strains investigated in this study will be named accordingly.

## Material and Methods

### Genome sequencing

The TeHV3 strain US1976/98 was grown on Terrapene heart cells (TH-1;ATCC-CCL 50 Sub-line B1; American Type Culture Collection, Rockville, MD, USA), harvested and pelleted as previously described [36]. Total DNA was extracted using the DNAeasy kit (Qiagen Hombrechtikon, Switzerland). A total of 3.5μg of viral DNA was delivered to a biotechnology company (Fasteris SA, Geneva, Switzerland) for next generation sequencing (NGS). The viral DNA was processed using the Illumina technology for NGS. Briefly, the DNA was fragmented to produce short DNA inserts to build a DNA template library with each of the inserts carrying universal adaptors. Following quality control, the library was sequenced with the Illumina HiSeq 2000 to obtain 50 base long single-end reads. *De novo* genome assembly was performed using the VELVET software (http://www.ebi.ac.uk/~zerbino/velvet/). To optimize the assembly, several values of k-mers were tested. To evaluate the quality of the assemblies, the following indicators were computed: sum of the contigs (DNA consensus sequences obtained by overlapping shorter DNA sequences) lengths, number of contigs, N50 (the length of the smallest contig in the set that contains the fewest (largest) contigs whose combined length represents at least 50% of the assembly) [41], number of contigs to reach the N50, average and maximum lengths of the contigs, and the number of reads from the library that can be mapped on the contigs. The assembly with the highest N50 and percentage of mapped reads was then chosen.

Bridging of the contigs was performed using viral sequences obtained from two previously generated sub-genomic DNA libraries (*Hind*III and *Eco*RI) [10] [34]. Following the assembly, multiple PCRs spanning 1–7.5Kb were performed to assess the junctions between contigs and the overall correctness of the assembly of the whole genome. Sequencing of the PCR products was carried out when the amplicon obtained was not consistent with the expected size derived from the sequenced genome. Finally, the single-end Illumina reads were mapped to the obtained genome sequence using BWA (Burrows-Wheeler aligner (http://bio-bwa.sourceforge.net)) [42]; the remapping was visualized with IGV (Integrative Genomic Viewer 2.3.34) [43]. For open reading frame (ORF) identification the program “ORF finder” (http://www.ncbi.nlm.nih.gov/projects/gorf/) was used and each putative ORF was manually assessed using the program “BLAST” (http://blast.ncbi.nlm.nih.gov/Blast.cgi). The gene identity was attributed according to the best hit provided by BLAST. An arbitrary cutoff corresponding to predicted genes encoding for at least 90 amino acids (aa) was selected when no homology could be found with any other known herpesviral genes using BLAST. Detection of the tandem repeats was carried out using the software “Tandem repeat finder” [44], using standard settings.

#### Genome comparison

The TeHV3 genome was compared with that of *Chelonid herpesvirus 5* (ChHV5), the only other available genome of a chelonian herpesvirus [22] using the program EasyFig v2.1 [45] with standard settings. Briefly the annotation files of the two genomes (HQ878327-ChHV5 and KT008627-TeHV3) were converted by the software into the corresponding tBLASTX files [45]. The graphic output of the comparison was then generated. Color tones from light gray to black were selected for the direct matches spanning from low to high, respectively, and orange to red for the inverted matches spanning from low to high, respectively.

### Animals and pathology

A retrospective investigation was carried out on selected formalin-fixed, paraffin embedded (FFPE) tissues obtained from ten tortoises necropsied from 1999 to 2009 and stored in the archive of the Centre for Fish and Wildlife Health (FIWI) of the University of Bern, Switzerland. All the tortoises selected for the study were captive animals and were either diagnosed as presumptively infected with TeHV or carrying compatible lesions based on having at least one of the following criteria: 1) eosinophilic to amphophilic intranuclear inclusions in at least one of the examined tissues (= presumptively infected); 2) presence of diphtheronecrotic stomatitis and/or glossitis (= compatible lesion); and 3) pneumonia (= compatible lesion). All these tortoises died of natural causes, with the exception of Z02/1970 that was humanely euthanized by the attending clinician because of very poor prognosis with an overdose of sodium pentobarbital performed by intra vascular injection. None of these tortoises was included in experimental studies. Two additional tortoises (PN186/12, PN13/08), selected using the above criteria, were provided by the University of Milan (Italy) along with a third tortoise (PN191/12) that despite absence of consistent lesions was also included in the study because it came from the same die off as PN186/12. A tortoise with a diphtheronecrotic stomatitis (S12/1458) submitted for necropsy to the Institute of Animal Pathology (ITPA) of the University of Bern was also included in the study. The three tortoises provided from the University of Milan and the tortoises submitted to the ITPA also died of natural causes and were not part of any transmission study. Finally, a tortoise (TG4/1998) experimentally infected with the TeHV3 type-strain US1976/98 with well-characterized associated pathology [34] was also considered for this study. This tortoise was part of a transmission study carried out previously. The study was approved by the Institutional Animal Care and Use Committee (IACUC) of the University of Florida and the tortoise was humanely euthanized with an overdose of sodium pentobarbital performed by intracoelomatic injection [34]. All the tissues available from all the tortoises were reviewed by two of us (FCO and MT). The following data was obtained for each tortoise: 1) time of death; 2) sex; 3) species; 4) age; 5) geographical origin 6) presence of any inflammatory, necrotic or degenerative lesions in any of the sections observed using light microscopy, and presence and location of the intranuclear inclusions. No live animals were included in this study. A detailed list of the tortoises included in this study including the identification numbers of each animal is provided in Table 1.

**Table 1:** Tortoise and TeHV strains.

Tortoise ID	TeHV Strain ID	Species	Year	Month	From	TeHV Diagnosis	Weight	Sex	Main pathology findings
Z02/1970	CH1970/02	*T*. *hermanni*	2002	April	CH	Negative	1Kg	F	Glossitis
Z03/1690	CH1690/03	*T*. *hermanni*	2003	March	CH	Negative	2.1Kg	F	Stomatitis
Z07/1611	CH1611/07	*T*. *hermanni*	2007	April	CH	Positive	0.37Kg	F	Glossitis**,pneumonia, meningoencephalitis
Z06/2360	CH2360/06	*T*. *hermanni*	2006	June	CH	Positive	0.39Kg	M	Stomatitis and glossitis**
Z01/2053	CH2053/01	*Testudo*. *sp*	2001	April	CH	Positive	1.15Kg	N/A	Glossitis**, lymphoid depletion, pulmonary edema and splenitis
Z08/5132	CH5132/08	*T*. *hermanni*	2008	December	CH	Negative	0.02Kg	F	Pneumonia, enteritis, lymphoid depletion, nephritis, esophagitis and epicarditis
Z00/7204	CH7204/00	*S*. *pardalis*	2000	December	CH	Positive	2.7Kg	M	Glossitis**, hepatitis, enteritis**, pneumonia**, meningitis, tracheitis and vasculitis**
Z03/6883	CH6883/03	*T*. *hermanni*	2003	October	CH	Positive	0.9Kg	M	Glossitis**, stomatitis, hepatitis, pneumonia**, meningitis, perivasculitis
Z07/2313	CH2313/07	*T*. *hermanni*	2007	June	CH	Positive	0.13Kg	F	Stomatitis, glossitis**, lymphoid depletion
Z01/3429	CH3429/01	*T*. *hermanni*	2001	June	CH	Positive	0.09Kg	M	Glossitis**, hepatitis, pneumonia, Ttacheitis, rhinitis, lymphoid depletion and necrosis
S12/1458	CH1458/12	*T*. *hermanni*	2012	March	CH	Positive	0.346Kg	M	Stomatitis**, lymphoid depletion
TG4/1998*	US1976/98	*T*. *graeca*	1998	August	USA	Positive	0.8Kg	M	Stomatitis (experimental infection)
PN186/12	IT186/12	*T*. *hermanni*	2012	N/A	IT	Negative	0.07Kg	M	Tracheitis, nephritis, gastritis
PN191/12	IT191/12	*T*. *graeca*	2012	N/A	IT	Negative	0.115Kg	F	Ovarian neoplasia
PN13/08	IT13/08	*T*. *horsfieldii*	2008	Fall	IT	Positive	N/A	N/A	Pneumonia**, tracheitis, enteritis, stomatitis

N/A = Not available

* = This tortoise was euthanized during a previous study.

** = Lesions with intranuclear inclusions.

Year and months refer to the time of death of the tortoises.

TeHV Diagnosis = Positive or negative cases of herpesvirus infection based on the presence or absence, respectively, of the characteristic intranuclear inclusions in stained tissue sections. TG4/1998 is the only exception since although no inclusions were observed in the examined tissues, the tortoise was known to be infected with TeHV3 given that was part of a group of tortoises experimentally infected with US1976/98 [34].

### Virus isolation

Virus isolation was attempted from fresh tissues obtained from tortoises PN186/12 and PN191/12 (from the University of Milan, Italy) and from an oral swab from tortoise S12/1458 (from the ITPA of the University of Bern, Switzerland). Tissues and swabs were processed for viral isolation by inoculation onto TH-1 cells at 28°C as previously described [34]. Cell cultures were monitored daily for the detection of cytopathic effects (CPE). The type-train 1976/96 was already available from previous studies [34, 36].

### TeHV3 strain detection and partial characterization of the gB gene

Total DNA was extracted either from infected cell cultures (strains 1976/96, CH1458/12 and IT191/12) or from FFPE tissue blocks containing at least a section of the tongue and/or oral mucosa or lung from all the remaining tortoises in the study. Three 20 μm sections were obtained from each of the selected paraffin blocks and placed into an RNAse/DNAse-free 1.5 ml Eppendorf tube. Total DNA was extracted from each set of paraffin slices with the FFPE DNA extraction kit (Qiagen Hombrechtikon, Switzerland). Total DNA was also extracted from infected cell cultures as previously described [34] (**Origgi et al., 2004**) and quantified with a Nanodrop spectrophotometer (Thermo Scientific, Wilmington, DE, USA). The complete gB gene of strains US1976/98, CH1458/12 and IT191/12 was amplified by PCR with a forward (gBTeHV3FW) and a reverse (gBTeHV3RV) primers (Table 2) which were designed on the basis of the sequence available from the US1976/98 genome (KT008627). The PCR reaction-mix contained 5 μl of 2 μM forward and reverse primers, 1.25 μl of each 10 mM dNTPs (Promega, Madison, WI, USA), 5 μl of 10x PCR buffer, 1 μl of PFU II Ultra DNA polymerase (Agilent Technologies, Santa Clara, CA, USA) 250 ng of total DNA template and double distilled water (Promega, Madison, WI, USA) up to 50 μl. PCR reactions were carried out in a DNA engine thermal cycler (MJ Research, Waltham, MA, USA) and comprised an initial denaturation step at 95°C for 2 minutes, followed by 40 cycles comprising a denaturation step at 95°C for 30 seconds, an annealing step at 50°C for 30 seconds and an elongation step at 68°C for 1 minute followed by a final elongation step at 68°C for 10 minutes. Given that FFPE tissues undergo DNA fragmentation, no full amplification would have been possible with a single PCR reaction. Consequently, either full or partial gB gene amplification from the paraffin blocks-derived DNA was carried out with a series of sequential and overlapping PCR reactions, which were set up with each of the subgroup of primer sets listed in Tables 2 and 3, respectively. More specifically, the pairs FWA and RVA, FW1 and RVB, FW2 and RVC, FW3 and RVD, FW4 and RVE, FW5 and RVF, FW6 and gBTeHV3RV were used to amplify several DNA fragments covering the full length gB of strain CH6883/03, while the primer pairs FW4 and RVE, FW5 and RVF, FW6 and gBTeHV3RV were used to amplify the 3’ half portion of the gB gene of all the other paraffin-blocks-derived strains in the study. Each of the PCR reaction mixes contained 0.25 μl of 10 μM forward and reverse primers, 12.5 μl of hot-start master mix (Qiagen AG, Hombrechtikon, Switzerland), 250 ng of total DNA template and double distilled water (Promega, Madison, WI, USA) up to 25 μl. The reaction mixes were placed in a DNA engine thermal cycler (MJ Research, Waltham, MA, USA) and after an initial denaturation at 95°C for 15 minutes (hot start), 45 cycles comprising denaturation at 95°C for 1 minute, annealing at 50°C (FWA and RVA, FW4 and RVE, FW5 and RVF) or 52°C (FW1 and RVB, FW2 and RVC, FW3 and RVD, FW6 and gBTeHV3RV) for 30 seconds, an elongation at 72°C for 30 seconds, followed. A final step comprising 10 minutes extension at 72°C was added. A summary of the primer pairs used for the gB gene amplification and cycling conditions is listed in Tables 2 and 3.

**Table 2:** PCR and sequencing primers.

Primer name	Primer sequence (5’-3’)
FWA	TTTGGTTATCATATTAGGAGCG
RVA	ACGTCTATTATTTCGTCACGC
FW1	GAGCATAGGTCATAGAACAACTATACG
RVB	GTCCTCGTTCATAGTTTCGG
FW2	AGATGTCTCCATTCTACGATAGAACC
RVC	CAGATGAGTATCCTTGTACCG
FW3	CCGACCGAAGGCAAAAAAGAAATAGA
RVD	AAACGCGACGCTTCATTATGG
FW4	ACAATTAAATAAAATAAATCCC
RVE	ATATACGTGCTTACTTCTGGG
FW5	ATAATTTTGTTAGGATGGTTC
RVF	TAAAATGATAGTAAATCCTCC
FW6	TTTTTCAGTAACCCGTTCGGAGG
gBTeHV3FW	AAAATGATCATGTGGTTATCGTT
gBTeHV3RV	AAAATTATTGGGAGGAATCGTCTATCT

**Table 3:** Glycoprotein B gene PCR amplification conditions.

Glycoprotein B amplification conditions			
gB amplification	Primer sets	Amplicon size (nt)	Cycling conditions
Full (from fresh tissues)	gBTeHV3FW-gBTeHV3RV	2,484*	95°C for 1’ + 40 cycles = 95°C for 30”, annealing at 50°C for 30” + 68°C for 1’. Final elongation step at 68°C for 10’
Full (from formalin-fixed tissues)	FWA-RVA	524*	95°C for 15’ (hot start)+ 45
	FW1-RVB	409*	cycles = 95°C-1’ + annealing
	FW2-RVC	413*	at 50°C (FWA and RVA,
	FW3-RVD	409*	FW4 and RVE, FW5 and
	FW4-RVE	405*	RVF) or 52°C (FW1 and
	FW5-RVF	389*	RVB, FW2 and RVC, FW3
	FW6-gBTeHV3RV	387*	and RVD, FW6 and
Partial	FW4-RVE	405*	gBTeHV3RV) for 30”, +
	FW5-RVF	389*	72°C for 30”. A final
	FW6-gBTeHV3RV	387*	elongation step for 10’ at 72°C

* Expected amplicon size including primers

### Partial amplification of the DNApol gene

The partial sequence of a highly conserved portion of the TeHV3 DNApol gene, accounting for 5% of the entire DNApol gene was carried out as previously described [33].

### Sanger Sequencing

Sequencing of the DNApol (partial sequence) and gB amplicons (partial and full length gB gene) was performed with an automated sequencer ABI Prism 3100 genetic analyzer (Applied Biosystems, Foster City, CA, USA) with the BigDye Terminator cycle sequencing kit (Applied Biosystems, Foster City, CA, USA). The primers used for the sequencing reactions were the same to those used to obtain the amplicons, respectively (see Tables 2 and 3). Sequencing of the partial DNApol gene amplicons was performed with the primer set recommended by Vandevanter et al. [33].

### Phylogenetic analyses

Multiple phylogenetic analyses were carried out. The first analysis (**1**) was based on the partial aa sequence of the DNApol and gB proteins, respectively, encoded in the genome of the TeHVs strains included in this study (N = 15). These sequences were compared to homologous sequences available from other herpesviruses (TeHV1-AB047545.1, TeHV2-AY916792.1, TeHV4-GQ222415.1, ChHV5-AF239684.2, ChHV6-EU006876.1 and GaHV1- AF168792.1 for the DNApol; and ChHV5-AAU93326, ChHV6-AAM95776 and GaHV1-YP_182356 for the gB sequences). The second analysis (**2**) was based on the partial nt sequences of the gB gene of all the TeHV3 strains included in this study (n = 15), which were compared to the homologous gene of other herpesviruses (ChHV5-AAU93326, ChHV6-AAM95776 and GaHV1- YP_182356). The third analysis (**3**) was carried out using the complete aa sequence of the gB protein of the reference strain (US1976/98), which was compared to homologous sequences of 36 other herpesviruses. Sequence alignment was carried out using multiple software packages (ClustalW, ClustalW2, TCoffee, Mafft L and Muscle) [46–49] for phylogenetic analysis **1**, whereas ClustalW was used for analysis 2 and 3. Maximum likelihood phylogenetic trees were obtained using MEGA6.0–6 [50] software package with 500 bootstrap replications, Jones Taylor Thorton replication method, uniform rates among sites and very strong branch swap filter.

### Recombination analysis

Assessment of recombination events of the gB gene was carried out with the Recombination Detection Program (RDP4) (Version 4.46) [51] using standard settings and feeding the software with two of the predicted parent strains (US1976/98 and CH6883/03) together with the predicted recombinant strain (CH3429/01). The methods used for the recombination assessment were those included in the software package and included RDP, GENECONV, BootScan, MaxChi, Chimera, SiScan, PhylPro, LARD and 3Seq, all operating under standard settings.

## Results

### Sequence analysis of the TeHV3 genome

**Fig 1:**
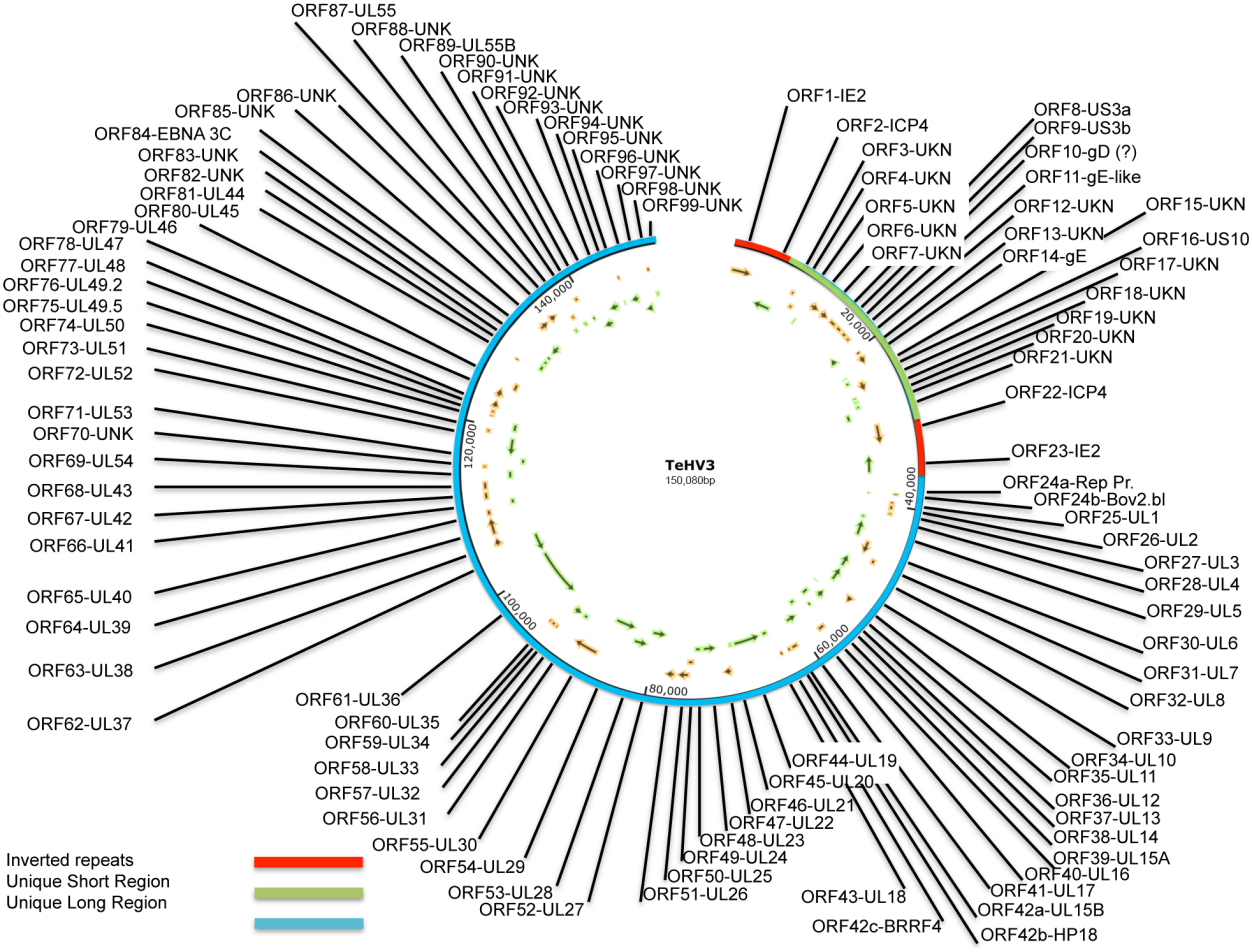
TeHV3 genome map. The TeHV3 genome is composed of a unique long region (UL) (blue bar) and a unique short region (US) (green bar) flanked by two inverted repeats (red bars). The progressive numbers and the homologous herpesvirus genes of the identified sense (orange) and antisense (green) open reading frames (ORFs) are shown in the map (SnapGene Viewer-V2.7; www.snapgene.com).

Next generation sequencing (NGS): A total yield of 1.598 mega bases (Mb) was obtained with 31,959,585 clusters. 95.8% of the bases had a Q-score (measure of base-calling accuracy) larger than 30. The *de novo* assembly of the reads led to a total of 24 contigs spanning from 107 to 64,501 nt long and accounting for 140,195 nt (N50 of 23,603 nt). The average base coverage (depth) was 8,984. Bridging of the contigs was carried out by PCR and with the aid of the sequencing information derived from the *Hind*III and *Eco*RI subgenomic libraries as previously mentioned. The final sequence of the genome comprised 150,080 nt (Fig 1). The TeHV3 genome is composed of a unique long (UL) and a unique short (US) region, with the US flanked by inverted repeats consistent with a type D arrangement similar to that of *Human herpesvirus 3* (HHV3-VZV) and the recently described genome of ChHV5 [22]. The GC content was 45.8%.

#### Analysis of the sequence of the unique long (UL) region

The length of the available UL region was determined to be 112,838 nt (position 37,243 to 150.080 of the genome). The putative 5’ and 3’ ends of the UL region were identified as those regions immediately contiguous to the inverted and terminal repeats, respectively. The putative 5’ end of the UL region was obtained by NGS complemented with the sub-genomic library approach, whereas the sequence of the putative 3’ end was obtained by sequencing a PCR amplicon bridging the 5’ end region of the terminal repeat and the 3’ end of the UL region. This approach was attempted given that herpesvirus genomes are known to adopt a closed circle configuration within a few hours after the start of replication [52]. By harvesting the total DNA of early-infected TH-1 cells, we could successfully amplify the bridging regions between the terminal repeat and the 3’ end of the UL region. The actual putative 3’ end of the UL region was considered to be the last nucleotide prior to the beginning of the sequence corresponding to the reverse and complement of the 3’ end of the internal inverted repeat. Multiple PCRs confirmed the assembly obtained.

**Fig 2:**
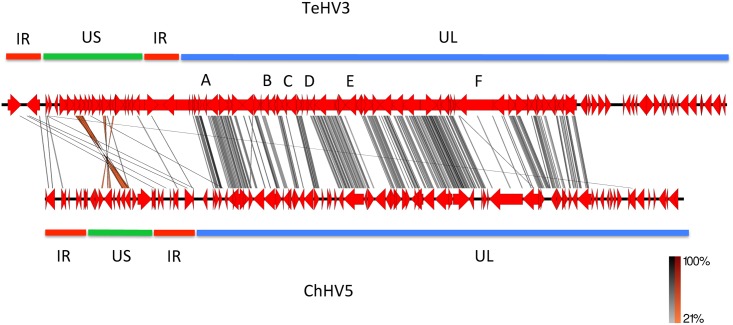
TeHV3 and ChHV5 genome comparison. The graphical comparison between TeHV3 and ChHV5 genomes obtained with the EasyFig1 software is shown in the figure. The genomes of TeHV3 and ChHV5 are depicted in red, with a black backbone. Similar regions are connected with gray (low) to black (high) (direct sequences) and orange (low) to red (high) (inverted sequences) bars. Green, blue and red bars highlighting the US and UL regions and of the inverted repeats (terminal and internal) of each genome, respectively are also shown. Letters A, B, C, D, E and F correspond to regions of the genomes that show no similarities within the conserved UL block.

The predicted UL region comprises at least 79 ORFs; 37FW, 42RV, with 35 partially overlapping. Sixty-two of them showed variable degrees of similarity with known herpesvirus genes, ranging from 38 to 79% for the UL47 (*Psittacid herpesvirus 1*) and UL 45 (*Ateline herpesvirus 3*) homologues, respectively. The remaining ORFs could not be associated unambiguously to a known herpesvirus gene (Table 4 and Figs 1 and 2).

**Table 4:** TeHV3 Open reading frames features.

ORF	HV Homologue	Protein	Function/ description	Similarity*	Position	Length (nt)	Frame
1	HHV6	IE2	Transactivator	40%77/188	1,097–3,916	2,820	+
2	ChHV5	ICP4	Transactivator	58%41/70	4,897–7,737	2,841	-
3	N/A	UNKN	N/A	N/A	8,948–9,580	633	+
4	N/A	UNKN	N/A	N/A	9,555–10,280	726	+
5	N/A	UNKN	N/A	N/A	10,704–11,444	741	-
6	N/A	UNKN	N/A	N/A	11,864–13,348	1,485	+
7	N/A	UNKN	N/A	N/A	13,041–14,798	1,398	+
8	ChHV5	US3a	Serine/Threonine Kinase	54% 181/333	14,902–15,993	1,092	+
9	ChHV5	US3b	Putative cyclin-dependent kinase 2	50% 153/305	16,026–16,964	939	+
10	ChHV5	gD	Cell receptor ligand	44%78/175	17,055–17,858	804	+
11	HPV2	gE-like	Unknown	41%58/141	17,874–18,542	669	+
12	N/A	UNKN	N/A	N/A	18,557–19,957	1,401	-
13	N/A	UNKN	N/A	N/A	20,069–20,512	444	+
14	ChHV5	gE	Host interaction and immunoevasion	41%159/387	20,634–22,031	1,398	+
15	N/A	UNKN	N/A	N/A	22,141–22,416	276	-
16	ChHV5	US10	Zinc-ion binding/ Tegument/capsid protein	54%106/194	22,683–23,429	747	-
17	N/A	UNKN	N/A	N/A	23,448–25,085	1,638	+
18	N/A	UNKN	N/A	N/A	25,127–25,489	363	-
19	N/A	UNKN	N/A	N/A	25,572–26,120	549	-
20	N/A	UNKN	N/A	N/A	26,268–26,969	702	-
21	N/A	UNKN	N/A	N/A	27,050–28,438	1,388	-
22	ChHV5	ICP4	Transactivator	58%41/70	29,506–32,346	2,841	+
23	HHV6	IE2	Transactivator	40% 77/188	33,327–36,146	2,820	-
24a	RFHVMn	Rep Protein	Unknown	38%34/88	38,525–38860	336	+
24b	BoHV6	Bov2.bl	Unknown	51%44/85	38,574–38,906	333	-
25	PsHV1	gL	Viral entry	58%33/56	39,402–39,856	384	+
26	ChHV5	UL2	UDG glycosilase	61%156/252	39,792–40,604	813	+
27	ChHV5	UL3	Phosphoprotein	72%154/211	40,685–41,266	582	+
28	ChHV5	UL4	Nuclear protein	50%86/172	41,682–42,365	684	-
29	ChHV5	UL5	DNA helicase/primase cpx	69%598/858	42,416–44,917,	2,556	-
30	ChHV5	UL6	Capsid protein	60%398/654	44,955–46,964	2,010	+
31	ChHV5	UL7	Virion egress protein	53%132/248	46,807–47,622	816	+
32	ChHV5	UL8	DNA helicase/primase cpx	44%336/749	47,627–49,777	2,151	-
33	AnHV1	UL9	Replication origin-binding protein	57%463/810	49,779–52,133	2,355	-
34	ChHV5	gM	Virion assembly and egress	66%279/418	52,145–53,383	1,239	+
35	HSV1	UL11	Tegument protein	62%27/43	53,433–53,672	240	-
36	ChHV5	UL12	Alkaline exonuclease	55%271/484	53,606–55,207	1,602	-
37	HSV1	UL13	Serine/threonine kinase	42%184/428	55,208–56,521	1,314	-
38	ChHV5	UL14	Tegument protein	58%58/100	56,443–56,976	534	-
39	ChHV5	UL15A	Putative DNA packing protein	64%214/330	56,975–58,090	1,116	+
40	ChHV5	UL16	Capsid binding protein	53%179/332	57,938–58,945	1,008	-
41	ChHV5	UL17	Virion Packaging protein	46%315/676	58,924–60,882	1,959	-
42a	ChHV5	UL15B	Terminase large subunit	74%267/359	60,986–62,050	1,065	+
42b	ChHV5	HP18	Hypothetical protein	43%34/79	62,069–62,523	453	+
42c	HHV4	BRRF4	BRRF4 protein	40%41/101	63,118–63,579	462	+
43	ChHV5	UL18/VP23	Capsid protein	65%211/321	63,784–64,746	963	-
44	ChHV5	UL19	Major capsid protein	72%993/1,373	64,821–68,930	4,110	-
45	ChHV5	UL20	Multifunctional essential for fusion	75%139/184	69,023–69,613	591	-
46	ChHV5	UL21	Tegument protein	45%220/480	69,716–71,068	1,353	+
47	ChHV6	UL22/gH	Fusion/cell entry	74%332/445	71,088–73,289	2,202	-
48	ChHV5	UL23/TK	De novo DNA synthesis	48%168/345	73,322–74,389	1,068	-
49	BoHV1	UL24	Nuclear protein	52%94/178	74,371–75,345	975	+
50	ChHV5	UL25	Virion packaging	55%323/578	74,960–756,687	1,728	+
51	ChHV6	UL26	Protease	60%352/579	76,726–78,198	1,473	+
52	ChHV6	UL27/gB	Cell entry/Fusion	73%637/864	78,251–80,734	2,484	-
53	ChHV5	UL28/ICP18.5	DNA processing and packaging	64%475/742	80,731–83,022	2,292	-
54	ChHV5	UL29/ICP8	Major DNA binding protein	63%735/1,156	82,976–86,527	3,552	-
55	ChHV5	UL30	DNA polymerase	70%809/1150	86,651–90,049	3,399	+
56	ChHV5	UL31	Nuclear matrix protein	67%185/275	89,991–90,878	888	-
57	ChHV5	UL32	DNA cleavage/packaging	63%296/466	90,884–92,497	1,614	-
58	ChHV5	UL33	DNA processing and packaging	66%72/108	92,478–92,825	348	+
59	ChHV5	UL34	Inner nuclear membrane protein	53%143/269	92,827–93,555	729	+
60	PsHV1	UL35	Small capsid protein	52%24/46	93,565–93,909	345	+
61	ChHV5	UL36	Large tegument protein	44%942/2101	93,906–101,630	7,725	-
62	ChHV5	UL37	Capsid assembly	43%461/1051	101,696–104,866	3,171	-
63	ChHV5	UL38	Capsid protein	56%256/455	104,868–106,211	1,344	+
64	ChHV5	UL39	Ribonucleotide reductase L.s.	62%490/787	106,315–108,705	2,391	+
65	FeHV1	UL40	Ribonucleotide reductase S. s.	73%220/301	108,728–109,642	915	+
66	ChHV5	UL41	Virion host shutoff protein	62%212/338	109,637–110,596	960	-
67	ChHV5	UL42	DNA polymerase processivity factor	48%147/304	110,732–111,838	1,107	+
68	ChHV5	UL43	Membrane protein	45%178/393	111,932–113,122	1,191	+
69	EqHV3	UL54/ICP27	Transactivator	50%109/214	113,152–114,438	1,287	-
70	N/A	UNKN	N/A	N/A	114,557–115,162	606	+
71	ChHV5	UL53/gK	Intracellular fusion	64%227/353	115,194–116,234	1,041	-
72	ChHV5	UL52	DNA primase	55%490/880	116,231–118,951	2,721	-
73	PsHV1	UL51	Tegument protein	47%82/174	118,950–119,600	651	+
74	MeHV1	UL50	Deoxyuridine triphosphatase	50%82/163	119,642–120,985	1,344	-
75	BoHV6	ORF53/UL49.5	Tegument/envelope protein	48%48/100	120,984–121,286	303	+
76	AnHV1	UL49.2	Tegument protein	60%59/98	121,352–121,990	639	+
77	SuHV1	UL48	Tegument protein	43%163/378	122,083–123,369	1,287	+
78	PsHV1	UL47	Tegument protein	38%95/247	123,467–124,888	1,422	+
79	AnHV1	UL46	Tegument protein	42%95/223	124,881–126,092	1,212	+
80	HHV3	UL45	Thymidylate synthetase	79%98/124	128,556–128,930	375	+
81	CeHV9	UL44/gC	Ligand/immunoevasion	45%104/228	128,974–130,026	1,053	-
82	N/A	UNKN	N/A	N/A	130,062–130,850	789	-
83	N/A	UNKN	N/A	N/A	130,954–131,490	537	-
84	HHV4	EBNA-3C	Nuclear protein	52%39/75	131,690–133,450	1,761	-
85	N/A	UNKN	N/A	N/A	133,338–134,915	1,578	+
86	N/A	UNKN	N/A	N/A	134,954–136,252	1,299	+
87	BoHV2	UL55a	Unknown	39%43/110	136,355–137,020	666	-
88	N/A	UNKN	N/A	N/A	137,152–137,841	690	+
89	ChHV5	UL55b	Unknown	44%28/63	138,014–138,670	657	-
90	N/A	UNKN	N/A	N/A	138,799–139,494	696	+
91	N/A	UNKN	N/A	N/A	139,517–140,005	489	-
92	N/A	UNKN	N/A	N/A	140,190–142,040	1,851	-
93	N/A	UNKN	N/A	N/A	142,037–143,752	1,716	-
94	N/A	UNKN	N/A	N/A	144,464–144,760	297	-
95	N/A	UNKN	N/A	N/A	144,780–145,166	387	+
96	N/A	UNKN	N/A	N/A	145,269–146,831	1,563	-
97	N/A	UNKN	N/A	N/A	146,976,-148,493	1,518	-
98	N/A	UNKN	N/A	N/A	148,510–148,968	459	+
99	N/A	UNKN	N/A	N/A	149,077149,700	624	-

*The number at the denominator indicates the total length of the portion of the herpesviral homologous protein being compared by BLAST with TeHV3. The number at the numerator indicates the actual number of the amino acid residues of TeHV3 showing similarities with the compared portion of the homologous protein. The percentage summarizes the overall similarity between TeHV3 and the specific herpesvirus homologous protein for the specific motif considered.

The UL ORFs are largely co-linear to the homologous genes of *Human herpesvirus 1* (HSV1), in particular from UL1 to UL43, while the segment spanning from UL53 to UL44 has an inverted orientation compared to HSV1. Two genes share homology with HSV1 UL55 (UL55a and UL55b), with 74% nt identities between each other, suggesting gene duplication. TeHV3 encodes for the thymidylate synthetase (UL45), whose homologue is found only in *Varicella zoster virus* (VZV) of all the *Alphaherpesvirinae*, whereas is common in *Gammaherpesvirinae*. The longest gene identified in the UL region was the homologous of HSV1 UL36, encoding for the large tegument protein. This gene is 7,725 nt long and encodes for a protein 2,574 aa long. In contrast, the shortest gene identified within the UL region is the homologous gene of HSV1 UL11. The gene is 240 nt long and encodes for a 79 aa long protein. Finally, upstream of the homologous gene of HSV1 UL1 is a 333nt ORF (ORF24b) encoding for a predicted 110 aa long protein that shares 51% similarity with a motif of a predicted protein with unknown functions encoded by *Bovine herpesvirus 6* (Table 4). Interestingly, almost entirely overlapping with ORF24b is ORF24a, which is another predicted ORF whose encoded predicted protein shares very limited similarities with a protein encoded by the retroperitoneal fibromatosis-associated herpesvirus (Genbank AGY30683) (Table 4).

Interestingly, the UL region of ChHV5, the closest related herpesviral genome to TeHV3, lacked several genes compared to TeHV3. More specifically, the HSV1’s UL13, UL40, UL44 through 51 and UL54 through UL56 homologous genes could not be detected in the ChHV5 genome [22]. In contrast, homologues of all these genes except for UL56 were identified in the TeHV3 genome. UL13 encodes for a serine-threonine protein kinase, whereas UL40 encodes for the small subunit of the ribonucleotide reductase. UL44 encodes for the glycoprotein C, a surface membrane protein which mediates immune evasion *in vivo* [53], while UL45 encoded for thymidylate synthetase. UL46, UL47, UL48 UL49.2, UL49.5 and UL51 encode for tegument proteins. UL50 encodes for a metabolic enzyme (dUTP diphosphatase). UL54 encodes for a transcriptional regulator (similar to ICP27), while UL55 encodes a protein with unknown functions. Ackermann and colleagues [22] reported that all the homologues genes from HSV-1 that are missing in ChHV5 in comparison with TeHV3 are known to be non-essential for herpesvirus replication *in vitro*. The biological meaning of these different sets of genes in TeHV3 is currently unknown. Table 4 provides a summary of all the comparison data.

#### Analysis of the sequence of the unique short (US) region

The US region was identified as the portion of genome between the terminal and inverted repeats. It is predicted to be 20,374 nt long (position 8,435 to 28,808 of the genome) and comprises at least 19 ORFs (11 FW, eight RV, two overlapping). Only five of the detected ORFs shared a relatively significant similarity with other known herpesviral genes (ORF8 and 9 with US3; ORF10 with gD; ORF14 with gE; ORF16 with US10) (Table 4). There are two genes that share high similarity with HSV1 US3, US3a (ORF8) and US3b (ORF9) (see below). ORF8 and 9 do not share any significant similarity between each other (only 10 positive aa between the two sequences), suggesting that an origin by gene duplication is unlikely. Fifty four per cent sequence similarity was observed between the TeHV3 ORF8 encoded protein and ChHV5 US3 protein homologue, whereas 50% sequence similarity was observed between the TeHV3 ORF9 encoded protein and the putative cyclin-dependent kinase 2 of ChHV5. The predicted protein encoded by the ORF 10 of TeHV3 shared 48% sequence similarity with a 175 aa long motif of ChHV5 glycoprotein D (gD), a molecule that is known to serve as the major receptor-binding protein in a number of *Alphaherpesvirinae* [22] (Table 4).

The longest predicted gene detected in the US region encoded for a protein of 545 aa, identified as TeHV3 ORF17, that did not share any similarity with any gene of known herpesviruses. The shortest ORF detected in the US region was instead identified as TeHV3 ORF15 and encoded for a 91 aa long protein and did not show any similarity with other known herpesvirus genes. The highest similarity between the identified proteins encoded by the TeHV3 US region and those of other herpesviruses was observed for the homologue of HSV1 US3 (see above) and of US10, both with 54% similarity with the correspondent protein encoded by ChHV5 (Table 4). Otherwise, the lowest similarity was observed for the glycoprotein E (gE) (ORF14) and of a gE-like protein (ORF11), with 41% similarity for the homologous proteins encoded by ChHV5 and *Cercopithecine herpesvirus 6*, respectively. No similarities could be found between the other TeHV3 US encoded predicted proteins and those of other known herpesviruses. Overall, findings were similar to those described for the US region of ChHV5, except for the number of ORFs identified, which in ChHV5 were 11 versus the 19 identified in TeHV3. Table 4 provides a summary of all the comparison data.

#### Analysis of the sequence of the inverted repeats (Internal and terminal repeats)

The regions identified as putative inverted repeats are both 8,434 long (position 1 to 8,434 and position 28,809 to 37,242 of the genome). The beginnings and the ends of the inverted repeats were identified as those regions immediately contiguous to unique sequences of either the UL or US regions. The inverted repeats encode for at least two predicted proteins with motifs sharing 58 and 40% similarity with two known transactivators, ICP4 (ORF2 and 22) and IE-2 (ORF1 and 23),(Table 4).

No detectable latency associated transcript (LAT) encoding gene was detected, in contrast with ChHV5. Strikingly, a major difference in the number of the predicted ORFs was observed between the inverted repeat regions of TeHV3 and that of ChHV5, where 12 ORFs were identified. However, while in ChHV5, the cutoff selected for the detection of the ORFs was 40 aa, we considered only the ORFs predicted to encode at least 90 aa as mentioned above (Table 4). Finally, five additional putative ORFs encoding more than 90 aa were actually identified in the TeHV3 inverted repeats, however, they were almost entirely clustered within the ICP4 homologous coding sequence and all of them showed very low similarities only with very short motifs of known herpesvirus genes and consequently were not considered in the final list of TeHV3 putative genes.

#### Tandem repeats

A total of 17 tandem-repeat regions were identified and the length of the repeat motifs ranged from 13 to 73 nt (Table 5). Four of them mapped within the UL region, eight in the inverted repeats, three in the US region, one bridging between UL region and the inverted repeat and one bridging between US and inverted repeat. In four of the repeated motifs up to 4 mismatches were observed. Two indels were also observed (Table 5).

**Table 5:** Tandem repeats features.

Tandem repeat sequence	Motif Length (nt)	Position	Location
CAGACTCCGTCCGTTAGATTTTGTCAAATTCTGGTCGAGTCGAAC	41	241–359	Inv. repeat
ACGTAACCCTAGCTGCTCTAAGGGACA	27	627–720	Inv. repeat
TGTCGGCCACCCTGACTCTACCCCGCTCCGGCCTGCCCCC	40	4015–4839	Inv. repeat
ATGCATATCATTAAATATGGAGGAGGTTATGGAATGAGGGAAGTCGGCTCCGATTGGTAGTTGGG	73	7949–8347	Inv. repeat
GGTGGATATGGGTGGATGTATGTTTATGCATATCATTAAAAAT* ^1^	43	8385–8563	Bridging 5’end US and inv.repeat
AAAAGCAAAACACTTGTATAAATTATCCAAT* ^2^	31	16943–17010	US3 region
GGTTGGGGAGAACGGTCATAATTAGAGGGACCG	33	27528–27703	ORF 21
TATCCGAGTTACTACCCCCCTGTACCTGCTGGAAAT* ^3^	36	27994–28312	3’end US
TTCCATAACCTCCTCCATATTTAATGATATGCATCTACCTGCCCCAACTACCAATCGGAGCCGACTTCCCTCA	73	28896–29294	Inv. repeats
CGGGGTAGAGTCAGGGTGGCCGACAGGGGGCAGGCCGGAG	40	32404–33228	Inv. repeats
GCTAGGGTTACGTTGTCCCTTAGAGCA	27	36523–36616	Inv. repeats
ATTTGACAAAATCTAACGGACGGAGTCTGGTTCGACTCGACCAGA	45	36884–37002	Inv. repeats
CCCTGGATCGGGCCC	15	38589–38844	ORF 24
ATATATAAATATA	13	62779–62971	UL15b/UL18 region
GACAACTTGAGGAGGAGGTGGTGGGTG	27	100095–100169	UL36
GGATCCTTAAACTAGATCCCTTTACATGTAGTATGTTACTATAAAAC	47	127865–128049	UL45/46 region
GGTGGGGTGATCGGAGGGGTAACCTCTTCT* ^4^	30	131704–131989	UL44 region

*Presence of mismatches or indels or both in the repeated motifs

^1^One mismatch

^2^Four mismatches and one indel

^3^One mismatch

^4^One indel

Inv. repeats = Inverted repeats

#### Ambiguous sequencing results

In the region comprised between the homologous genes to UL44 and UL55 we identified a tandem repeat region. The PCR bridging this repeated sequence yielded a product that appeared to be approximately 50 nt longer than the original predicted sequence. Several attempts to sequence that region failed. Similarly, the region across UL15b and UL18, which also contains repeated sequences, might be approximately 200 nt shorter than predicted by the original sequence.

The putative 3’ end of the UL region comprises a 5.4 Kb contig that was originally assembled in the opposite orientation of the one then determined to be the correct one. The ambiguity was determined by the conflicting results of a short bridging PCR over the sequence gap, which could not be confirmed with a long bridging PCR. The orientation of the contig presented in the final sequence here was selected on the basis of PCR and sequencing results. Similarly, between the IE-2 and ICP4 homologues we identified a GC rich region 1,015 nt long with repeated motifs (tandem repeats). Interestingly, the PCR product spanning between the two genes over the GC rich region showed multiple bands ranging from approximately 900 to 700 nt, but all smaller than the expected product. However, when running a PCR positioning the primers distally from the GC rich region, an amplicon of the expected size was obtained. The pattern of the short bridging PCR results was very similar to that described by Hommelsheim and colleagues [54] when sequencing repetitive DNA sequences.

In the context of a different research project, while screening subgenomic clones of TeHV3 strain US1976/98 obtained by partial digestion of the genomic DNA with *Sau3*AI we detected a repeated motif comprising alternating long series of single A, T, C or G (up to 22 nt long) that we could not find in the assembled genome. This repeated motif was contiguous with a sequence that was only partially matching that bordering a similar, but different repeated motifs at the beginning of the putative UL region. Blasting the novel identified nt motif (with the option “somewhat similar sequences” of BLAST) no match with any of the known herpesviruses was observed. Contrastingly, when the search was not restricted to herpesviruses only, identities up to 84% of portions of the sequence (up to 33%) with eukaryotic organisms (*Oryzias latipes*-HG313981.1) were observed.

Finally, the original sequence of the TeHV3 genome was determined to be 170 nt longer than the one presented in this article. This additional sequence was located at the 5’ end of the terminal repeat and it was one of the two motifs of a tandem repeat originally identified in that region. When assessing the assembled genome by the software IGV, a total of 12 ambiguous nt were observed across the entire genome. Of these, six clustered in the first 222 nt of the genome and two were in the last 20 nt of the genome. In particular in association with four of the nt ambiguities at the 5’ end of the genome (in correspondence of the 5’ end of the terminal repeat), we also observed an abrupt drop of the coverage. Interestingly this drop in coverage corresponds to the joining of the two tandem repeats described above (region between original nt 166 to 172). Multiple attempts of sequencing these regions were carried out, but the presence of repeated motifs did not allow us to conclusively resolve these ambiguities. Given the clustering of several sequence and assembly ambiguities in the region across these two tandem repeats and that once the two tandem repeats were collapsed into just one, the assembly of that region appeared to be more robust, we considered that this 170 nt fragment might have represented a sequencing artifact and then it was removed. Following the editing of the sequence according to what described above, the inverted and tandem repeats, which originally differed in size for exactly 170 nt in length, were then both measuring exactly 8,434 nt, further supporting the editing described above. A possible insertion of a nt was instead detected at position 127,377, whereas a possible deletion of a nt was observed at position 131,553 of the genome. A drop of coverage of 30% between contiguous nt was seen at positions 37,191 and 37,242 but the assembly in that region appears solid and no further assessment was considered necessary.

#### Genome Comparison

The graphic outcome of the software EasyFig1 (Fig 2), highlighted a prominent conservation of the arrangement of the genes clustering into the central portion of the UL region of the genome, although showing overall low similarity as suggested by the predominant light gray of the connecting lines. However, within this highly conserved portion of the genome, few regions showed absence of virtually any similarity. More specifically, the regions corresponding to the homologue of UL4 (A), UL12-13 (B), UL16 (C), in that between the UL15b and UL18 homologues (D) and in that corresponding to the UL22 (E) and UL36 (F) homologues, none or very limited numbers of connecting lines were observed. The extremities of the genomes showed a relative low or absence of similarities, with the most relevant clustering within the US regions of the genomes where low similarity-labeling color (orange) highlighted corresponding inverted matches (ORF 8-US3a, 9-US3b, 14-gE and 16-US10). No matches could be observed in the terminal portion of the putative UL regions of the two genomes.

### Animals

A total of 15 tortoises were included in this study. Ten of them were diagnosed as presumptively infected by herpesvirus and five as carrying consistent lesions (or history = IT191/12) based on the criteria described above. The only exception was tortoise TG4/1998, which although did not show any intranuclear inclusions in the examined tissues was also considered conclusively infected by TeHV given that it was part of a previous challenge study carried out with the TeHV3 strain US1976/98. Ten out of 15 were *T*. *hermanni*, one *T*. *horsfieldii*, two *T*. *graeca*, one *Testudo* sp, one *Stigmochelys* (formerly *Geochelone*) *pardalis*. The tortoises died either during the spring (April-June: n = 8) or the fall (October-December: n = 4) including also Z02/1970 that was euthanized because of very poor prognosis. One animal was part of a terminal transmission study and was euthanized in August. No information was available for two individuals. Seven were male and six female. No sex information was available for two tortoises. Most of the tortoises were from Switzerland (n = 11), fewer from Italy (n = 3) and one from the US, accounting for three countries and two continents. The selected cases spanned between 1998 and 2012. A complete summary concerning the tortoises’ information is provided in Table 1.

### Virus isolation

Cell cultures inoculated with tissue extracts from tortoise PN191/12 and swab washes from tortoise S12/1458 showed CPE consistent with cell rounding, detachment and cell lysis after 7 to 21 days and up to two blind passages performed. The presence of the virus in the supernatants of the cell cultures showing CPE was confirmed by PCR using the protocol described by Vandevanter and colleagues [33]. No virus could be isolated from the cell culture infected with the tissue extracts from tortoise PN186/12.

### Characterization of the TeHVs strains sequences

#### DNA polymerase (DNApol)

**Fig 3:**
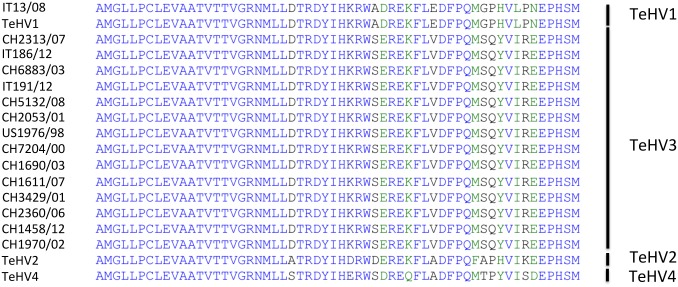
Alignment and comparison of the partial amino acid sequence of the DNA polymerase protein of the TeHVs strains. The alignment of the partial amino acid sequences of the DNA polymerase protein shows unique differences between the 4 recognized TeHVs genotypes. All but one of the detected TeHVs strains had sequences identical to TeHV3, whereas one strain belonged to genotype 1. (The Genbank accession numbers for TeHVs reference strains were: TeHV1-AB047545.1, TeHV2-AY916792.1, TeHV3 (1976/96 = US1976/98)-DQ343881, TeHV4-GQ222415.1. The partial DNA polymerase amino acid sequences of the TeHVs strains detected in this study could not be deposited in Genbank because their length was below the minimum required to be accepted)

Partial amplification of the DNApol gene from all the tortoises included in the study (N = 15) was carried out with consensus primers according to an established protocol [33]. The PCR products were consistent with the expected size (181nt) and the DNA sequences were translated into the predicted 60 aa long sequence (58 aa readable for strain CH5132/08). Fourteen out of the fifteen sequences shared 100% identity with the homologous sequence of other TeHV3 strains (DQ343881; TeHV3 1976/96 = US1976/98). The remaining strain (IT13/08) showed 100% identity with the homologous sequence of TeHV1 strains (AB047545.1). The aligned sequences along with those of TeHVs reference strains are shown in Fig 3.

DNApol aa sequence generated for the novel detected TeHV3 strains could not be deposited in the NCBI/Genbank database because their length was shorter than the minimum accepted.

#### Glycoprotein B (gB)

Within the portion of the genome identified as the UL region a 2,484 nt long ORF was determined to be the homologue of the HSV1 gB gene (UL27 = ORF52). The nucleotide sequence encoded for a protein 827 aa long. The protein was 25 aa shorter than the homologue of ChHV5 (852aa; Genbank AAU93326) and 38 aa shorter than that of ChHV6 viruses (865aa; Genbank AAM95776), the only reptilian herpesviruses with a complete available gB sequence.

**Fig 4:**
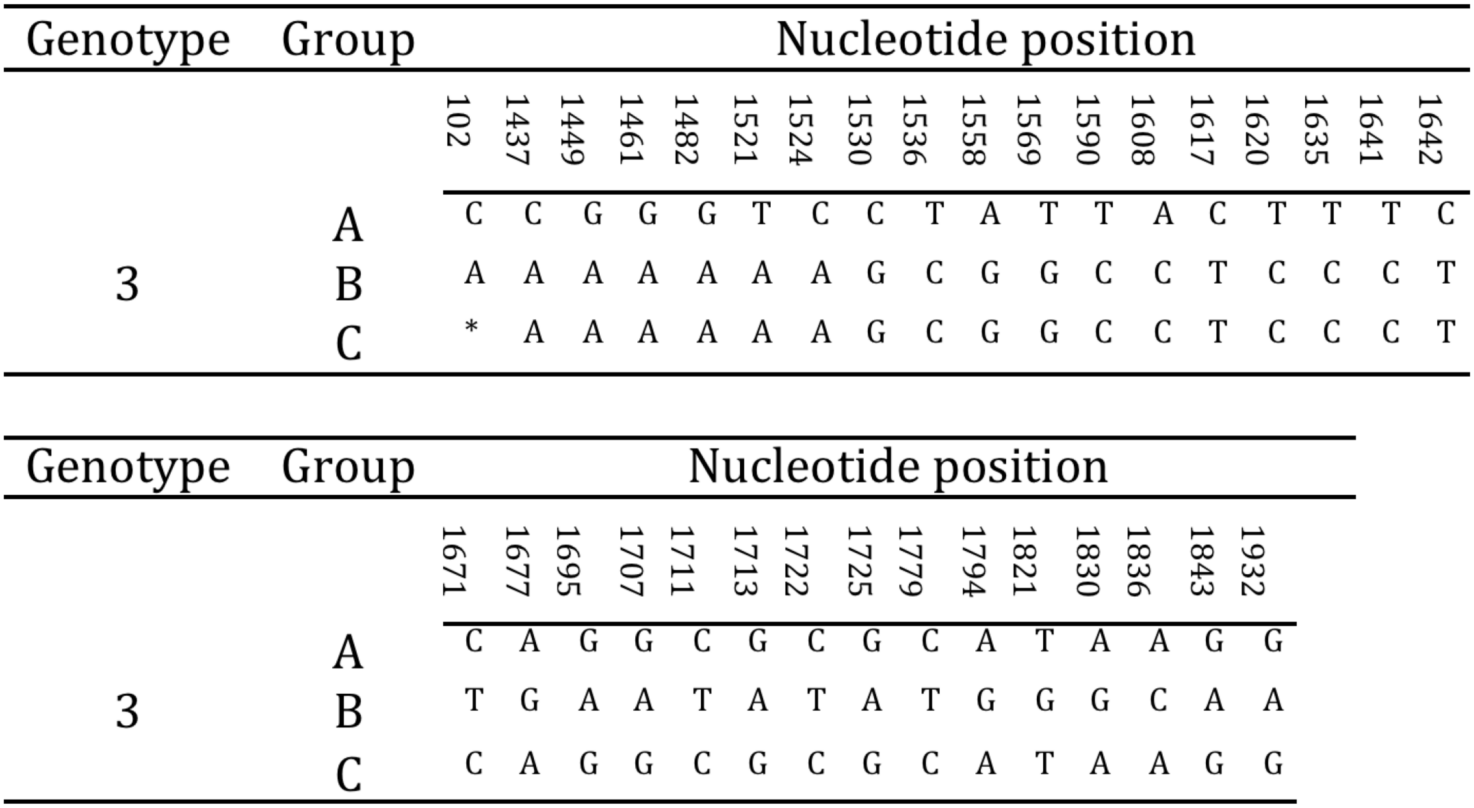
Hypervariable region of the gB gene of TeHV3. The unique nucleotide changes unambiguously differentiating the genogroup A, B and C are shown in the figure (* = not available nucleotide for the putative group C TeHV strain).

**Fig 5:**
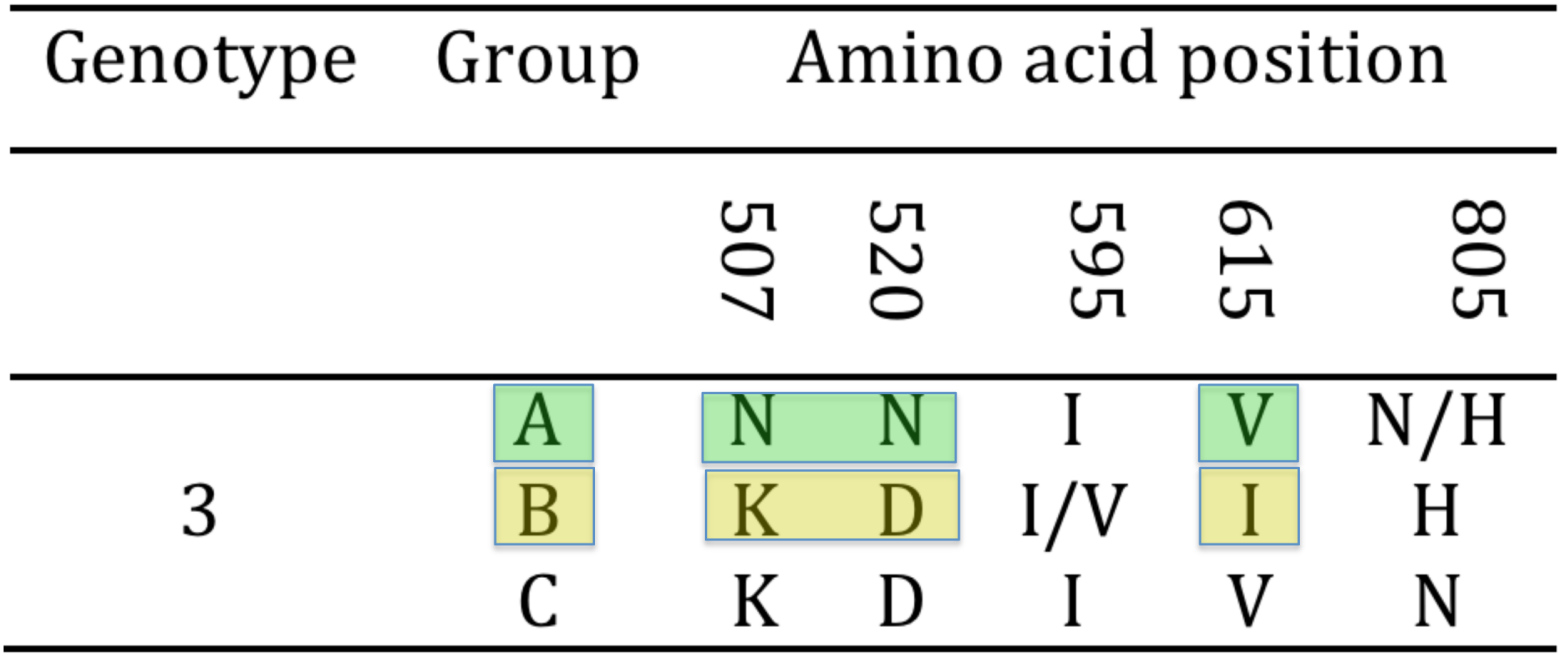
Hypervariable region of the TeHV3 gB protein. Three unambiguous amino acid changes differentiate the A and B genogroups within TeHV3 (colored blocks). The putative genogroup C shows an intermediate sequence between genogroup A and B.

**Fig 6:**
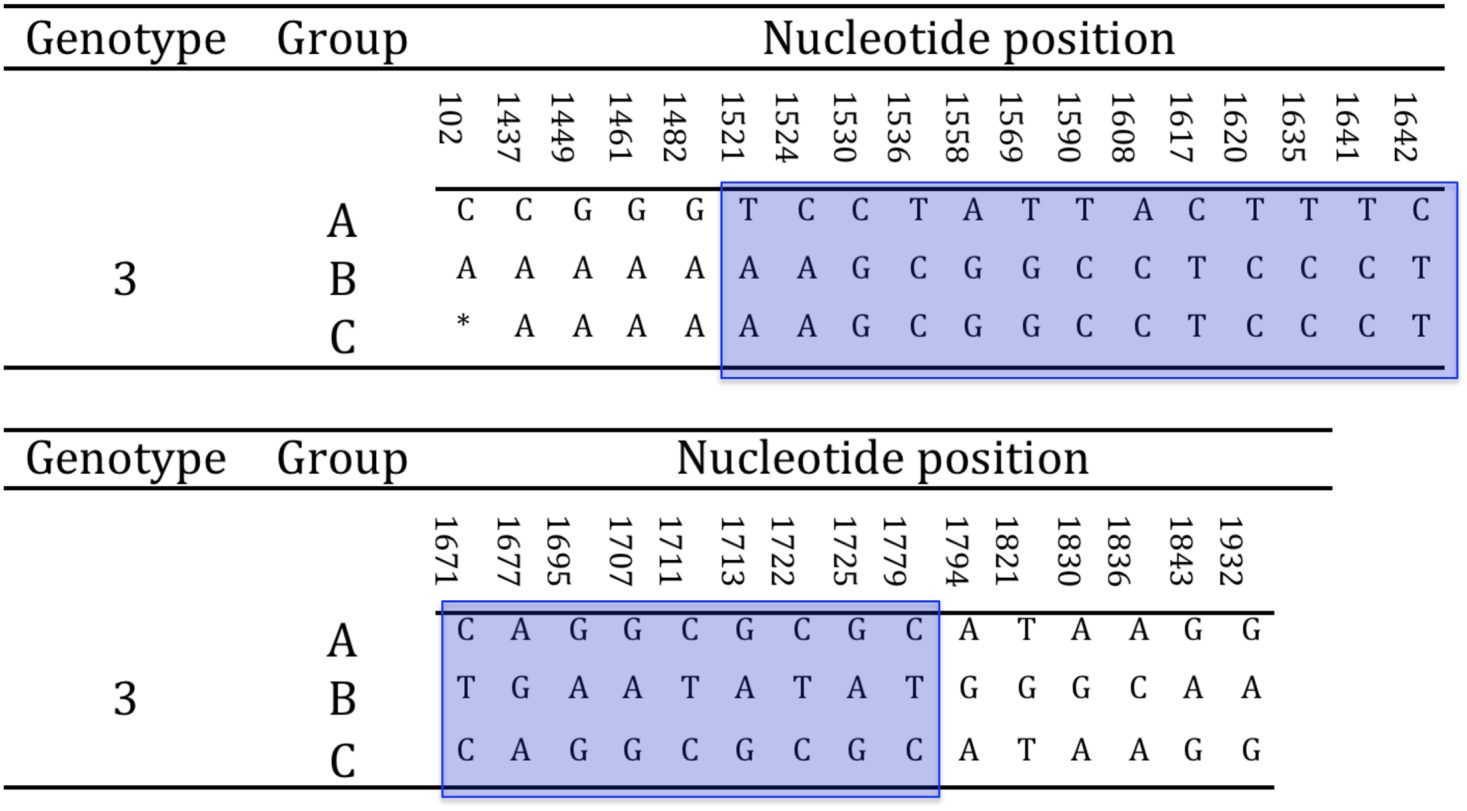
Selection of hypervariable nucleotide changes for phylogenetic analysis. In a hypervariable region of the glycoprotein B gene spanning across approximately 250 nt, are clustered 22 of the 33 unambiguous nucleotide changes differentiating the A and B genogroups within TeHV3. This region was selected for a high-resolution phylogenetic analysis of TeHV3 strains. The light blue block highlights the selected region of the gene. (* = not available nucleotide for the putative group C TeHV strain).

Either complete or partial amplification of the gB gene was successfully obtained for all tortoises in the study (N = 15). In particular, the full amplification of the gB gene was obtained for strains US1976/98, IT191/12 and CH1458/12, and CH6883/03 (Table 1). The sequences obtained showed the highest variability in the 3’half of the gene. The 3’ half partial sequence of the gB gene was then selected for phylogenetic analysis. The amplified portion of the gB gene was 1084 nt long (1069–1084 nt readable according to the amplicons, including the primers) and was obtained from all the remaining tortoises in the study (Table 1). The alignment of the complete and partial sequences of the gB genes of the different TeHVs strains revealed the existence of a total of 66 single nt polymorphisms (SNP) between two groups (genogroups) of strains (n = 11 and n = 3) named A and B, respectively. Two SNPs were located within the first 102 nt of the gB sequence, while the remaining 64 clustered in the 3’half of the gene. Furthermore, the strain CH3429/01 showed intermediate features between genogroup A and B and was identified as an additional putative group C. Of the 66 SNPs 33 were uniquely differentiating genogroup A from B (Fig 4). Five of the SNPs were missense, resulting in aa changes (Fig 5). All the missense SNPs clustered in the 3’ half of the gene. Within a 250 nt-long region of the highly variable portion of the gB gene were clustered most of the SNPs (22 SNPs; 1,521–1,779 nt) differentiating genogroup A and B; similarly for the additional putative recombinant strain (genogroup C) (Fig 6). This region of the gB gene was then selected as target region for TeHV3 genotyping.

**Fig 7:**
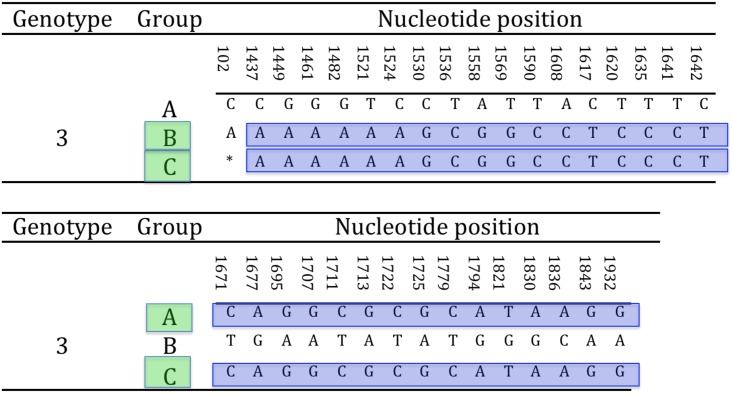
Glycoprotein B homologous recombination. The putative C genogroup shows full identity with either genogroup A or B partial gB nt sequence according to the portion of the hypervariable region considered. This arrangement is consistent with the outcome of homologous recombination. The highlighted sequence blocks show the homologous regions between the gB sequences of B and C and A and B genogroups, respectively (* = not available nucleotide for the putative group C TeHV strain).

The TeHV strain with intermediate SNPs between genogroup A and B (putative genogroup C) revealed a sharp regional demarcation of the SNPs arrangement. Briefly, the SNPs overlapping with those of genogroup A were located in the 3’ end of the hypervariable region of the gB gene, whereas the SNPs overlapping with those of the genogroup B were located in the 5’ end of the hypervariable region suggesting the occurrence of homologous recombination (Fig 7). The recombination event was confirmed by the software GENECONV, BootSCAN, MaxChi, Chimaera and 3Seq of the RDP4 package [51]. In particular, the MaxChi software identified the TeHV3 genogroup A as the most likely major parent and genogroup B as the most likely minor parent.

### TeHV DNApol- and gB-based phylogenetic analyses (1, 2 and 3)

**Fig 8:**
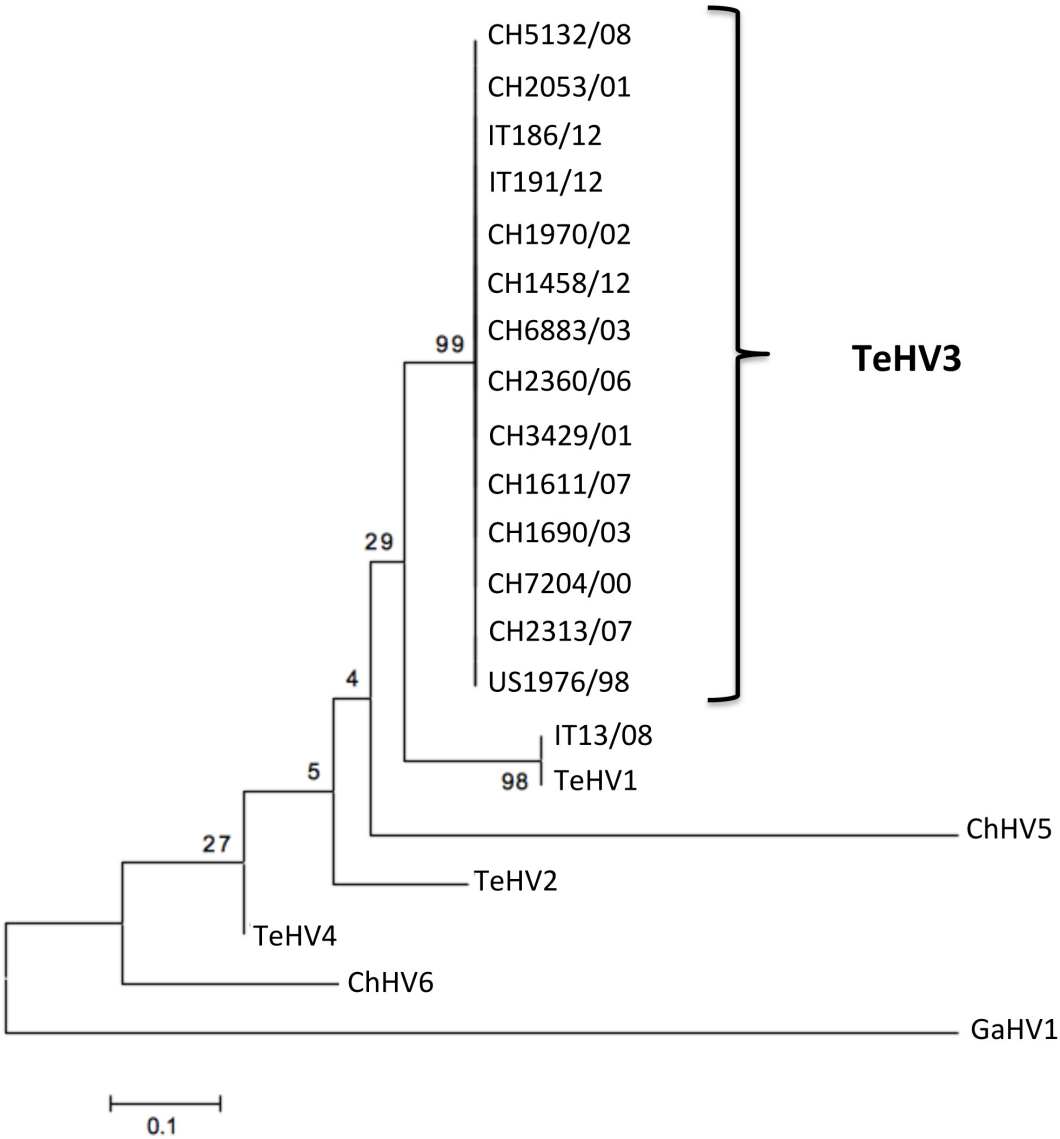
DNA polymerase-based phylogenetic analysis. Maximum likelihood tree inferred from the partial amino acid sequence of the DNA polymerase of the 15 investigated TeHVs strains and of the reference strains TeHV2, TeHV4, ChHV5, ChHV6 and Gallid herpesvirus 1 (GaHV1) that served as the outgroup. (Genbank accession numbers: TeHV1-AB047545.1, TeHV2-AY916792.1, TeHV3 (1976/96 = US1976/98) DQ343881, TeHV4-GQ222415.1, ChHV5-AF239684.2, ChHV6-EU006876.1, GaHV1- AF168792.1. No accession numbers could be obtained for the partial DNA polymerase protein sequences generated for the novel detected herpesvirus strains because their length was shorter than the minimum accepted by Genbank).

**Fig 9:**
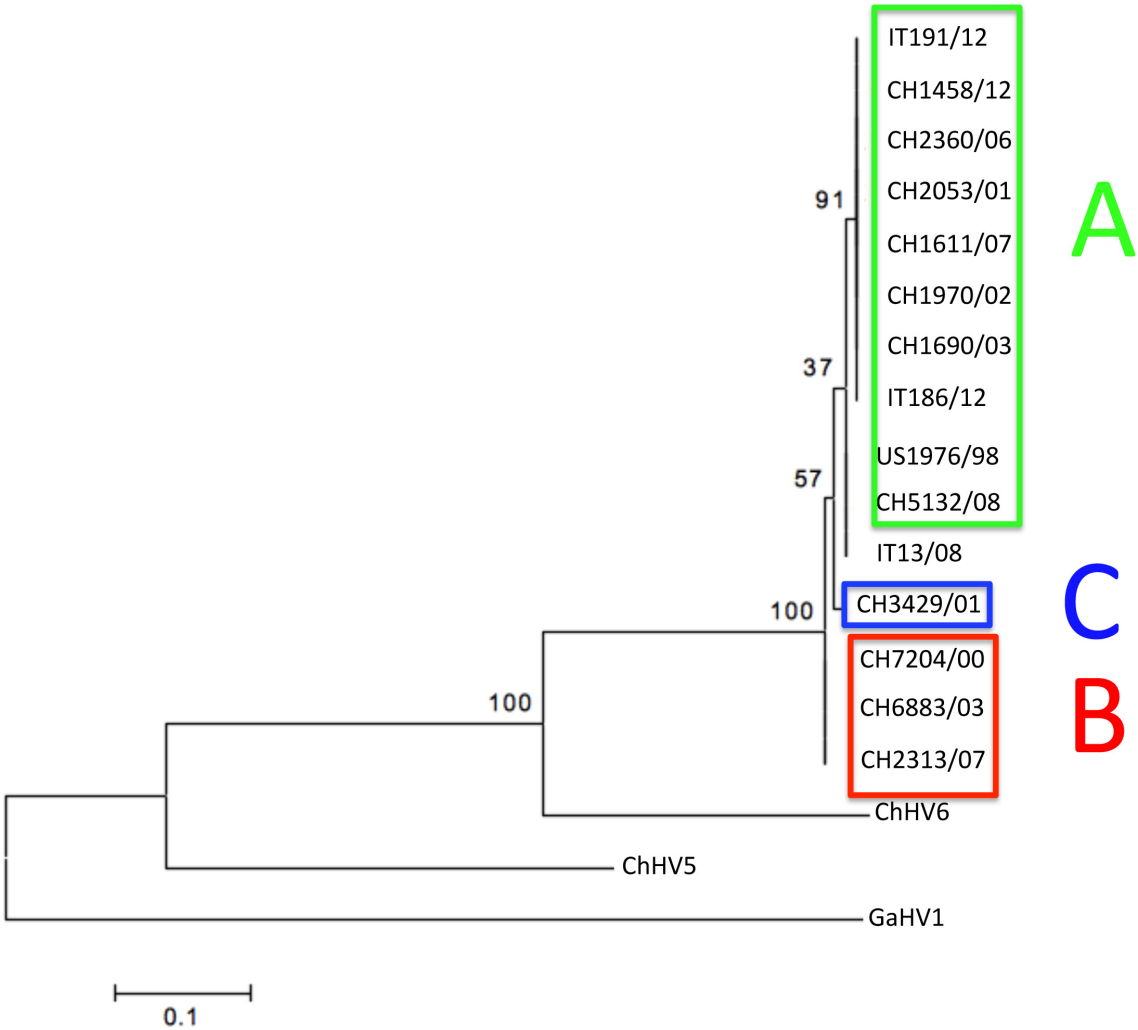
Glycoprotein B-based phylogenetic analysis. Maximum likelihood tree inferred from the partial aa sequence of the gB of the 15 TeHVs strains investigated in this study and of reference strains of other ChHV (ChHV5, -6). Gallid herpesvirus 1 (GaHV1) was included as outgroup. Bootstrap values from 500 iterations are shown. This analysis reveals the existence of at least two distinct genogroups within the TeHV3 genotype (A-green boxed and B-red boxed). A third putative genogroup (C-blue boxed) composed of a single strain is also observed. A TeHV1 strain (IT13/08) clusters within the A genogroup of the TeHV3 genotype. Clear separation of the TeHV3 genogroups from the sequences of other two chelonian herpesviruses (ChHV5 and ChHV6) is observed. (Genbank accession numbers: CH1970/02-KP979727, CH1690/03-KP979726, CH1611/07-KP979721, CH2360/06-KP979725, CH2053/01-KP979723, CH5132/08-KP979729, CH7204/00-KP979719, CH6883/03-KP979730, CH2313/07-KP979722, CH3429/01-KP979720, CH1458/12-KP979718, US1976/98-KP979717, IT186/12-KP979728, IT191/12-KP979716, IT13/08-KP979724, ChHV5-AAU93326, ChHV6-AAM95776, GaHV1-YP_182356).

The phylogenetic analysis carried out on the partial aa sequences of the DNA pol (Type 1 analysis) revealed the presence of 14 strains clustering within TeHV3 and one within TeHV1 genotypes, respectively (Fig 8). In contrast, the phylogenetic analysis based on the partial aa sequence of the gB protein (Type 1 analysis) revealed the existence of two distinct TeHV3 genogroups supported by significant bootstrap values (Fig 9). Additionally, the strain CH3429/01 clustered alone in an intermediate position between the two main TeHV3 genogroups paralleling the findings of the gB sequencing described above. The partial gB aa sequence of the single TeHV1 strain, IT13/08, clustered together with the TeHV3 genogroup A strains (Fig 9). Overlapping results were obtained independently from the alignment software used.

**Fig 10:**
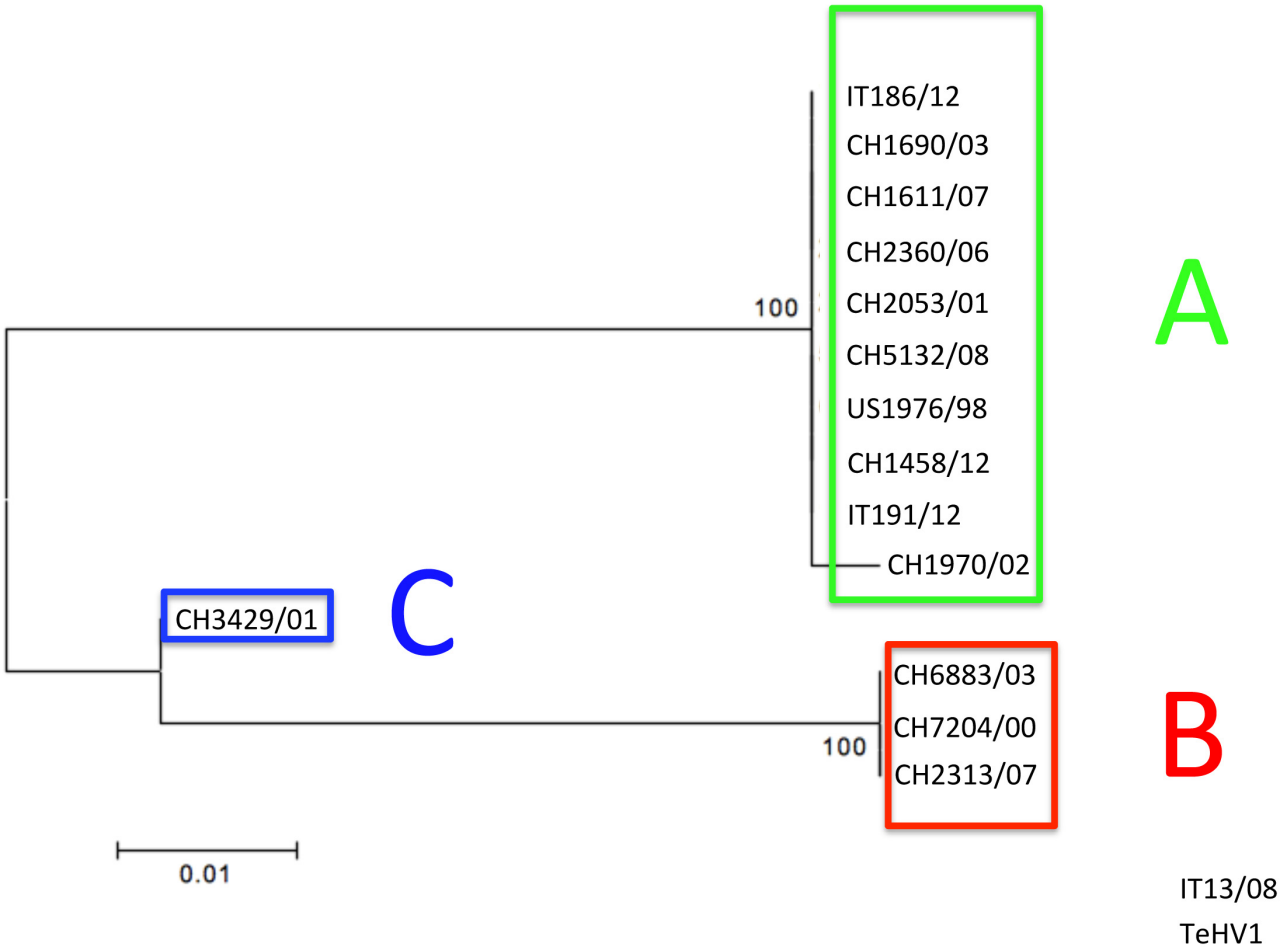
Glycoprotein B hypervariable region-based phylogenetic tree. The phylogenetic analysis (Maximum likelihood tree) based on the hypervariable region of the glycoprotein B gene unambiguously identifies two main genogroups (A-green boxed and B-red boxed) and a third minor one (C) (blue boxed) within the TeHV3 genotype (Genbank accession numbers: CH1970/02-KP979727, CH1690/03-KP979726, CH1611/07-KP979721, CH2360/06-KP979725, CH2053/01-KP979723, CH5132/08-KP979729, CH7204/00-KP979719, CH6883/03-KP979730, CH2313/07-KP979722, CH3429/01-KP979720, CH1458/12-KP979718, US1976/98-KP979717, IT186/12-KP979728, IT191/12-KP979716, IT13/08-KP979724).

The separation between the genogroups A and B was even more robust when the phylogenetic analysis was carried out using the 250 nt of the hypervariable region of the gene described above (Type 2 analysis) (Fig 10).

**Fig 11:**
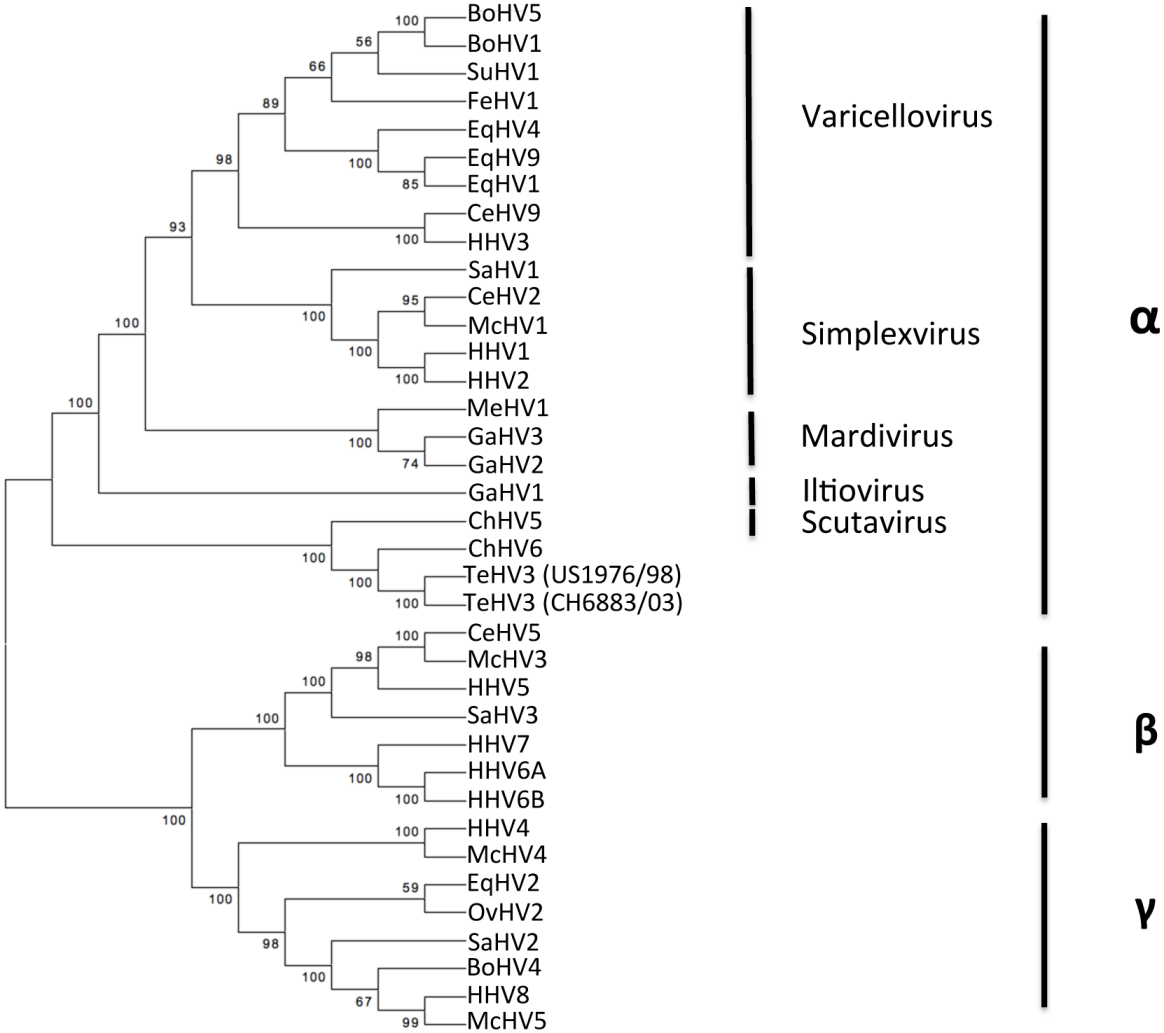
Glycoprotein B (full sequence)/based phylogenetic analysis. Maximum likelihood tree inferred from the full-length aa sequence of the gB of 2 strains of the TeHV3 and of 35 additional herpesviruses. Bootstrap values from 500 iterations are shown. This analysis shows unambiguous clustering of the TeHV3 main genogroups (A and B) within the *Alphaherpesvirinae* subfamily in close association with ChHV5 member of the novel identified genus of the *Scutavirus*. (Genbank accession numbers: Chelonian HV5-ChHV5-AAU93326, Chelonian HV6-ChHV5-AAM95776, Bovine HV5-BoHV5-YP_003662497.1, Bovine HV1-BoHV1-NP_045331.1, Suid HV1-SuHV1-YP_068330.1, Feline HV1-FeHV1-YP_003331552.2, Equine HV4-EqHV4-NP_045250.1, Equine HV9-EqHV9-YP_002333514.1, Equine HV1-EqHV1-YP_053078.1, Cercopithecine HV9-CeHV9-NP_077446.1, Human HV3-HHV3-NP_040154.2, Saimirine HV1-SaHV1-YP_003933812.1, Cercopithecine HV2-CeHV2-YP_164470.1, Macacine HV1-McHV1-NP_851887.1, Human HV1-HHV1-NP_044629.1, Human HV2-HHV2-NP_044497.1, Meleagrid HV1-MeHV1-NP_073321.1, Gallid HV3-GaHV3-NP_066859.1, Gallid HV2-GaHV2-YP_001033956.1, Gallid HV1-GaHV1-YP_182356.1, Testudinid HV3-TeHV3-CH6883/03-KP979730, Testudinid HV3-TeHV3-US1976/98-KP979717; Cercopithecine HV5-CeHV5-YP_004936031.1, Macacine HV3-McHV3-YP_068182.1, Human HV5-HHV5-YP_081514.1, Saimirine HV3-SaHV3-YP_004940228.1, Human HV7-HHV7-YP_073779.1, Human HV6A-HHV6A-NP_042932.1, Human HV6B-HHV6B-NP_050220.1, Human HV4-HHV4-YP_001129508.1, Macacine HV4-McHV4-YP_068009.1, Equine HV2-EqHV2-NP_042604.1, Ovine HV2-OvHV2-YP_438135.1, Saimirine HV2-SaHV2-NP_040210.1, Bovine HV4-BoHV4-NP_076500.1, Human HV8-HHV8-YP_00119354.1, Macacine HV5-McHV5-NP_570749.1).

Finally, when the full gB aa sequence was compared with the homologous sequence of other well-characterized herpesviruses, TeHV3 clustered unambiguously among the *Alphaherpesvirinae* in close association with ChHV5, the only recognized herpesviral species of the genus *Scutavirus* (Type 3 analysis) (Fig 11).

### TeHV3 genogroups A and B appear to be associated with distinct lesional patterns

Pathological examination was performed on all the 15 tortoises in the study. A summary of the pathological findings is available in Table 1. Briefly, of all the tortoises infected with TeHV3 genogroup A strains (n = 10), four had inclusions limited to one tissue and no inclusions were detected in any tissue from the remaining tortoises infected with this TeHV3 genogroup. Differently, all the tortoises infected with TeHV3 genogroup B (n = 3) showed inclusions in at least one tissue and two tortoises had inclusions in more than one tissue. Pneumonia was observed both in tortoises infected with TeHV3 genogroups A and B, but it was overrepresented in the tortoises infected with TeHV3 genogroup B (two out of three). In contrast, in tortoises infected with TeHV3 genogroup A, pneumonia was seen in a minority of individuals (two out of eight with available lung tissue). Furthermore, inclusions were seen in the lung of both TeHV3-genogroup-B-infected tortoises with pneumonia, while no inclusions were seen in the affected lungs of tortoises infected with TeHV3 genogroup A. Of the tortoises infected with TeHV3 genogroup A for which brain tissue was available (n = 6) only one showed tissue changes consistent with meningitis. In contrast, meningitis was seen in two out of the three tortoises infected with TeHV3 genogroup B. Vasculitis and/or perivasculitis were observed in two out of three individuals infected with the TeHV3 genogroup B. The tortoise infected with the *bona fide* recombinant TeHV3 strain had inclusions in the tongue and developed pneumonia. The only tortoise infected with a TeHV1 strain had pneumonia, tracheitis and hepatic lipidosis. Inclusions were seen only in the lung (Table 1).

### Phylogeography of TeHV3 genogroups in Switzerland

**Fig 12:**
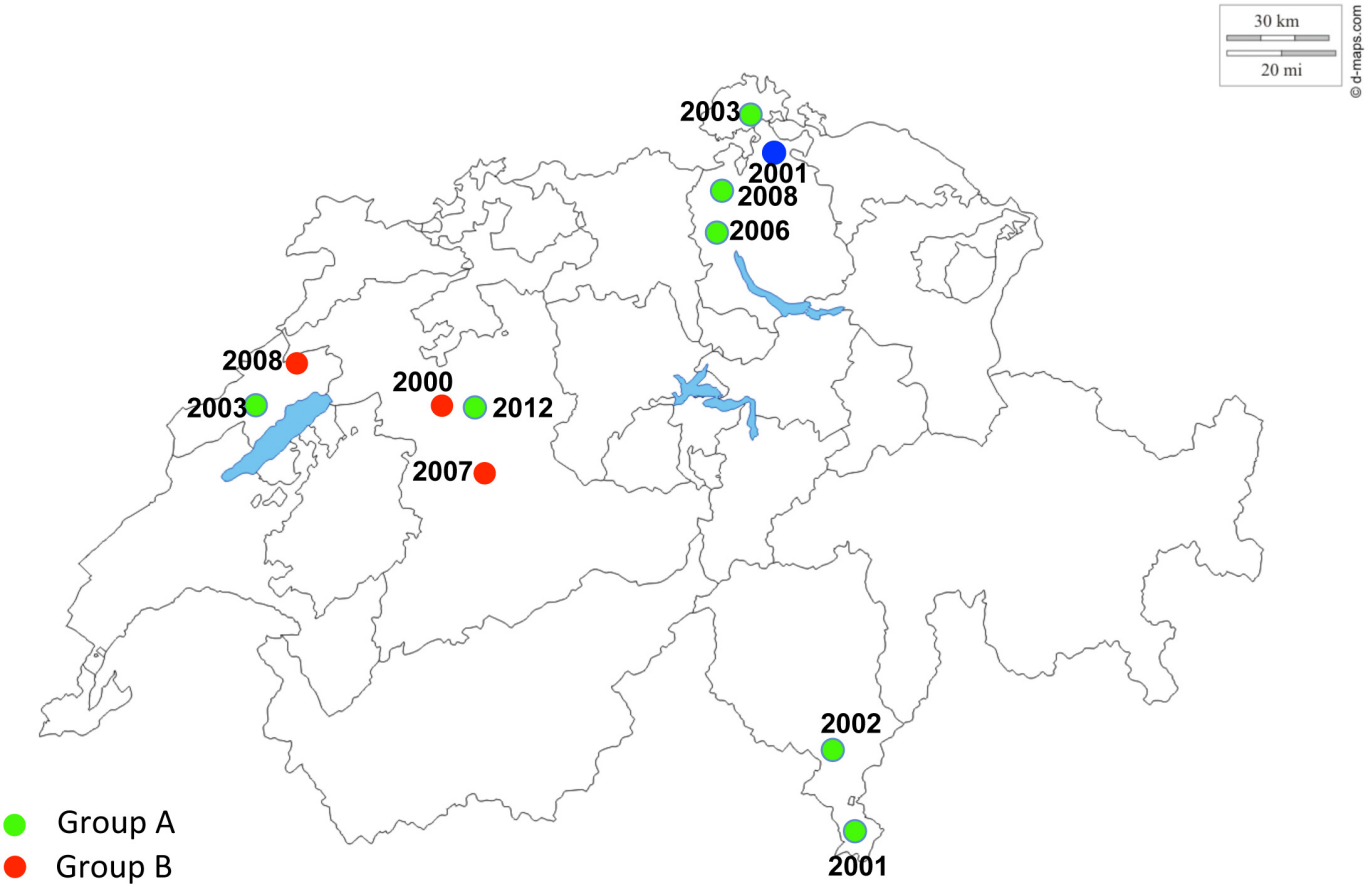
TeHV3 phylogeography (Switzerland). Green dots show the geographic origin of the tortoises infected with TeHV3 genogroup A, whereas red dots correspond to the geographic origin of the tortoises infected with TeHV3 genogroup B. The blue dot shows the location of the only tortoise infected with the recombinant strain originated from putative homologous recombination between a strain from the genogroup A and one from the genogroup B. The year next to the respective color dot correspond to the time when the specific tortoise died of TeHV3 infection. Main borders of Switzerland and of the Swiss cantons are also shown in the figure along with the main lakes (http://d-maps.com/carte.php?num_car=24787&lang=de).

The distribution of the infected tortoises from Switzerland was closely examined given that they were the most numerous and representative group. Fig 12 summarizes the locations of the tortoises infected with different TeHV3 strains. Tortoises infected with either genogroups A or B or the recombinant strain (C) were already present on the Swiss territory in 2000/2001 and additional cases of tortoises infected with either A or B genogroups were observed during the following years, at least up to 2008. In contrast, no additional infections with the recombinant TeHV3 strain were recorded (Fig 12).

## Discussion

The main goal of this research project was to fill a major gap concerning the biology of one of the most relevant viruses of reptiles, thereby providing new data to further investigate and better understand host-pathogen interactions in chelonians. This first example of a *de novo* assembly of a chelonian herpesvirus revealed that the TeHV3 genome shares several common features with other known *Alphaherpesvirinae* including length, gene-content and overall arrangement.

The only other chelonian herpesvirus genome available to date is that of ChHV5. The two genomes share the overall arrangement and show the most similarities within the UL region. This is consistent with the findings of Alba et al. (2001) [55], with the most conserved gene blocks in the *Herpesviridae* spanning from the UL5 to the UL39 homologues. Despite the similarities between the two chelonian virus genomes, interesting differences were identified. For example the two genomes appear to differ by approximately 18 Kb in length, with ChHV5 lacking several genes that were identified in TeHV3 [22]. The missing genes encode for proteins that apparently are dispensable for viral growth *in vitro* [22]. Among these are UL 46, 47, 48, 49.2, 49.5 and 51, which encode for structural elements such as tegument proteins. The absence of these genes might be compensated by other genes with similar functions that are present in the ChHV5 genome. However, other genes present in TeHV3 and absent in ChHV5 might have more critical roles for TeHV3. These include genes encoding for enzymes such as seronine-threonine kinase (UL13) that is likely relevant for intracellular signaling and cell cycling, and a dUTP diphosphatase (UL50), which together with the small subunit of the ribonucleotide reductase (UL40) and the thymidylate synthetase (UL45) are important for *de novo* synthesis of DNA. UL 54 encodes for the homologous gene of HSV1 ICP27 a potent gene transactivator. Additionally, the lack of UL44 homologous gene encoding for the glycoprotein C (gC) in ChHV5 in comparison to TeHV3 might be critical for immune evasion. Glycoprotein C is known to bind to C3 and to inhibit virus neutralization [53]. The overall functional implications associated with the presence or absence of these genes in TeHV3 and ChHV5, respectively, are difficult to predict without the appropriate investigations. However, the lack of genes encoding for proteins relevant for the *de novo* synthesis of viral DNA and subsequently for viral replication, together with that of a major immediate early gene such as the homologue of ICP27 might help to explain some of the different features of TeHV3 and ChHV5, including for example the failure to grow ChHV5 in cell culture or the distinct pathological changes caused by these two viruses: necrotizing lesions in tortoises infected with TeHV3 and proliferative lesions of neoplastic nature in sea turtles infected with ChHV5. Further *in vitro* and *in vivo* studies are necessary to explore the functional bases for these differences.

The differences in gene content of TeHV3 and ChHV5 may be secondary to differences in co-evolution that might have occurred with their respective hosts. Chelonians are a group of vertebrates that has evolved into multiple families that have diverged over millions of years [56], with adaptations to very different environments such as dry land and oceans. The genus *Testudo* comprises several species commonly infected by TeHV3. This genus is part of the family *Testudinidae*, which includes tortoises, dry land chelonians whose appearance in the fossil record dates back to the late Cretaceous [57]. Tortoises of the genus *Testudo* are considered to have originated in the African continent and later spread into Europe [56]. *Cheloniidae* is one of the two families of sea turtles having members susceptible to infection with ChHV5. Of the *Cheloniidae*, *Chelonia mydas* and *Caretta caretta* are two species of sea turtles most commonly infected by ChHV5. Their ancestors, the *Americhelydae* are believed to have originated in the North American continent during the Cretaceous [58], whereas their presence in the Mediterranean is considered to be more recent and occurred probably no more than 12,000 years ago [59]. No detection of ChHV5 has been yet reported in Mediterranean sea turtles. This suggests that it is unlikely that TeHV3 and ChHV5 might have diverged recently from a hypothetical common ancestor consistently with the results of the genome comparison (Fig 2). The presumptive long-standing independent co-evolution of the two viruses with hosts with distinct anatomical and physiological adaptations to their native habitats, may partially account for the different content in genes between TeHV3 and ChHV5. More sequencing data including that of TeHV2, the only North American TeHV genotype known to date are needed to contribute to better understand the actual correlation and evolutionary relationships between *Testudinid herpesviruses* and ChHV5. Finally, the presence in TeHV3 (and the absence in ChHV5) of the homologous gene of UL45, which encodes for the thymdylate synthetase gene present only in HHV3-VZV among the *Alphaherpesvirinae* but common in *Gammaherpesvirinae* was an interesting finding. However, despite this common feature with a *Varicellovirus*, TeHV3 does not appear to cluster in this genus. Furthermore, TeHV3 clusters closely to ChHV5, a *Scutavirus*.

The novel genomic information described in this report is considered an important step to further illuminate the host-pathogen interactions in chelonians. The sequencing of the TeHV3 genome has allowed for the identification of several homologous genes to those of well-characterized herpesviruses. Among these, the gB gene is one of the most critical genes for herpesvirus infectivity given the role of its encoded protein in cell entry [39, 40]. Furthermore, gB is both relatively well conserved among herpesviruses and at the same time it is under a likely higher evolutionary pressure than the DNApol because of the direct pressure exerted by the host immune system eliciting the production of neutralizing antibodies [40]. These features convinced us to select the gB gene sequence (and translated aa sequence) as an ideal phylogenetic marker to attempt to increase the phylogenetic resolution power among the TeHV3 strains and potentially trace correlations between distinct TeHV3 genogroups and pathology phenotypes in tortoises. The phylogenetic analysis performed with the partial sequence of gB, showed unambiguously the existence of at least two distinct genogroups of TeHV3 strains, named A and B, respectively, which could not be detected when using the partial DNA pol sequence. The phylogenetic analysis performed on a highly variable region of the gB gene further highlighted this distinction. The localization of four of the five missense substitutions spanning across a relatively central portion of the gene (1521-1845nt; Figs 5 and 6) is of interest given that it does not have a correspondence in the homologue gene of the type species of *Alphaherpesvirinae*, HSV1. Comparing different HSV1 gB aa sequences (data not shown), aa changes occur either within the very proximal N- and C-portion of the protein. The crystal structure of HSV1 gB has recently become available and antigenic and mutational analysis suggests that several domains spanning for most of the length of the protein ectodomain are required for virus entry [39]. The different clustering of aa changes occurring in TeHV3 gB might suggest a different arrangement of the most functionally relevant domains of TeHV3 gB than the HSV1’s homologue. Similar distribution of the missense mutations is observed in ChHV5 strains. However, the specific functions of the different HSV1’s gB domains are not yet fully understood and more investigations are needed to understand potential functional differences between HSV1’s and TeHV3 gB.

Most of the identified strains in the fatally infected tortoises investigated in this study belonged to group A and a smaller number clustered within group B, possibly suggesting that subgroup A is the most common of the two genogroups. However, a large sample size is necessary to clarify this point. A distinct third group (C), which was identified in a single tortoise, was shown to be the result of homologous recombination between members of the A and B genogroups consistent with co-infection of tortoises with both TeHV3 genogroups. To the best of our knowledge this is the first example of homologous recombination demonstrated in a chelonian herpesvirus and the first indirect example of multiple TeHV3 strain infection in tortoises. The gB-based phylogenetic analysis (either with aa or nt sequences) clustered the only detected TeHV1 strain together with TeHV3 genogroup A, suggesting that the phylogenetic relationships between distinct TeHVs might be more complex than previously considered and might be influenced also by homoplasy. Consequently, our novel genotyping method is complementary to the one based on the DNA pol, and we recommend for a more thorough and precise TeHVs characterization the following hierarchical approach: 1) identification of the TeHV genotype by sequencing of the partial DNA pol gene [33] and 2) identification of the TeHV3 genogroups based on the gB gene sequence.

Within the limitations associated with the small sample size, we observed differences between the pathology phenotype associated with TeHV3 genogroups A and B infection. Specifically, TeHV3 genogroup B infection was associated with more frequent occurrence of intranuclear inclusions, viral pneumonia, vasculitis or perivasculitis and changes in the CNS than with TeHV3 genogroup A infection. These findings are suggesting of the existence of at least two subgroups of *Testudinid herpesvirus* responsible for different pathology. The putative recombinant strain between A and B subgroups showed intermediate pathogenicity between the two. However, since this is based on a single isolate, further isolates need to be studied to confirm this observation. A transmission study carried out with both TeHV3 genogroups will be necessary to confirm the observations described above.

An important contribution of this investigation is the indirect assessment of some anecdotal observations reported by veterinarians and tortoise breeders; which include tortoises developing clinical signs of herpesvirus infection primarily in the spring or late fall. These observations support circumstantial reports from veterinarians and tortoise breeders that die-offs of tortoises secondary to TeHVs infection mostly occur after the end or just prior to hibernation. Furthermore, a presumptive different sensitivity of different species of tortoises to TeHV infection and/or disease has been suggested for the Mediterranean Hermann’s and Greek tortoise [14]. Consistently, Hermann’s tortoises were overrepresented in our study group suggesting that this species is more sensitive to TeHV3. Further work is needed to substantiate these findings.

In conclusion, we have successfully performed the first complete *de novo* assembly of a chelonian herpesvirus, providing fundamental genetic information to obtain greater insight into the biology of this virus and the host-pathogen interaction in an early diverging vertebrate lineage. This enabled us to identify at least two distinct subgroups of the TeHV3 genotype and the distinct lesional profiles caused by these viruses in the tortoises examined in this study. This unearths new foundations for future studies on host-parasite resistance and infection.
